# Evaluating polymeric biomaterials to improve next generation wound dressing design

**DOI:** 10.1186/s40824-022-00291-5

**Published:** 2022-10-01

**Authors:** Jacob G. Hodge, David S. Zamierowski, Jennifer L. Robinson, Adam J. Mellott

**Affiliations:** 1 Bioengineering Graduate Program, University of Kansas, Lawrence, KS USA; 2 Department of Plastic Surgery, University of Kansas Medical Center, Kansas City, KS USA; 3 Department of Chemical and Petroleum Engineering, University of Kansas, Mail Stop: 3051, 3901 Rainbow Blvd, Lawrence, KS 66160 USA

**Keywords:** Biomaterials, Polymers, Tissue Engineering, Wound Dressings, Wound Healing

## Abstract

Wound healing is a dynamic series of interconnected events with the ultimate goal of promoting neotissue formation and restoration of anatomical function. Yet, the complexity of wound healing can often result in development of complex, chronic wounds, which currently results in a significant strain and burden to our healthcare system. The advancement of new and effective wound care therapies remains a critical issue, with the current therapeutic modalities often remaining inadequate. Notably, the field of tissue engineering has grown significantly in the last several years, in part, due to the diverse properties and applications of polymeric biomaterials. The interdisciplinary cohesion of the chemical, biological, physical, and material sciences is pertinent to advancing our current understanding of biomaterials and generating new wound care modalities. However, there is still room for closing the gap between the clinical and material science realms in order to more effectively develop novel wound care therapies that aid in the treatment of complex wounds. Thus, in this review, we discuss key material science principles in the context of polymeric biomaterials, provide a clinical breadth to discuss how these properties affect wound dressing design, and the role of polymeric biomaterials in the innovation and design of the next generation of wound dressings.

## Introduction to Biomaterials

**Fig. 1:**
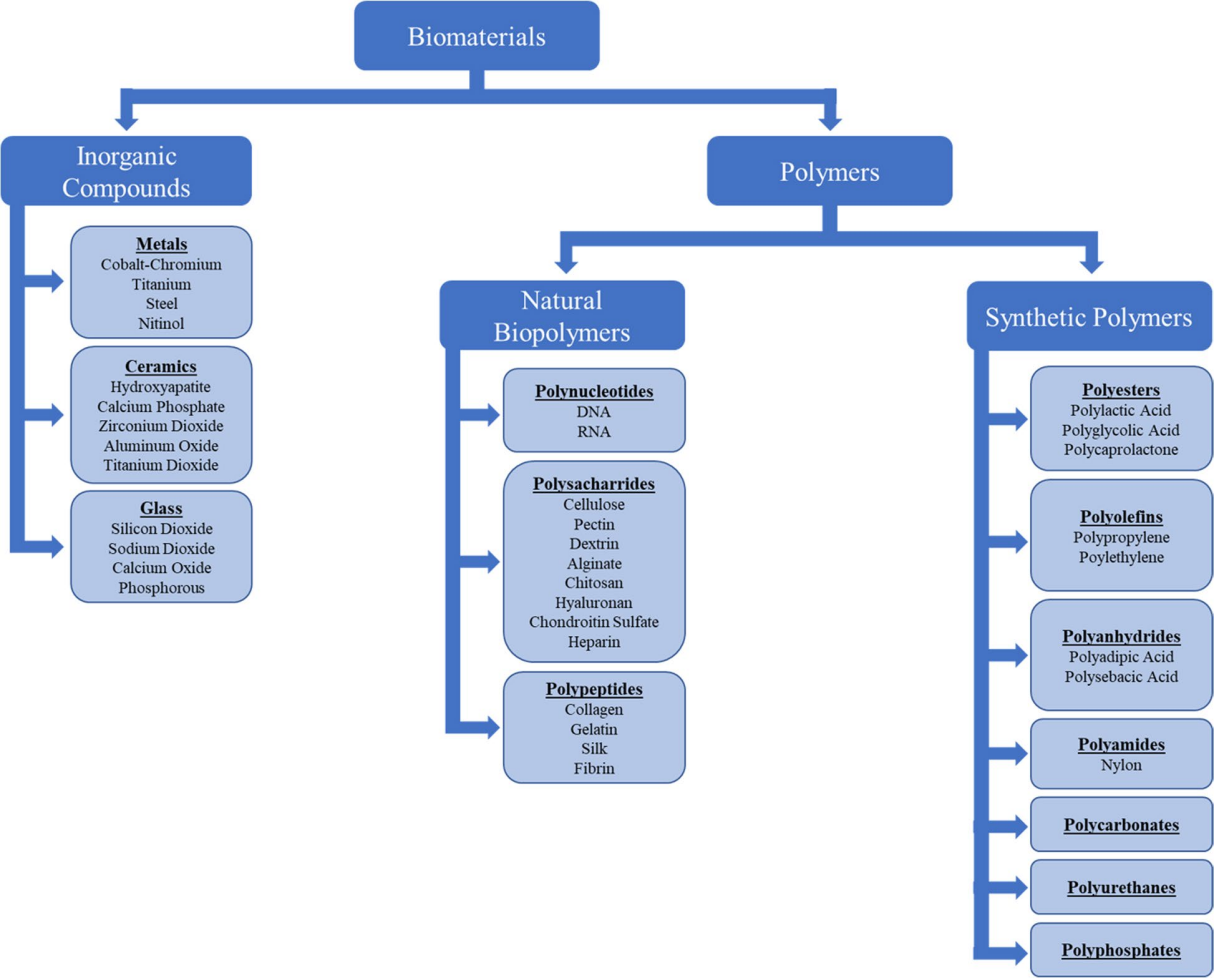
Flow chart of biomaterial classifications

Biomaterials can broadly be defined as any material substance that can be used as a diagnostic or adjuvant therapeutic system that aids in the detection or treatment of biologically derived ailments [1]. Consequently, biomaterials have become an essential aspect in the development of innovative therapies that have emerged from the field of tissue engineering over the last several decades, likely becoming a cornerstone in the future treatment of human disease [2]. Biomaterials are generally broken into three principal material classes, synthetic polymers, natural polymers, and inorganic compounds (Fig. 1), and can be used as an implantable or injectable system that permanently replaces a tissue defect, used to deliver biological compounds, or act transiently as a temporary matrix until the body is able to heal itself. Thus, biomaterials are exceedingly diverse in their composition, properties, and ultimate ability to modulate tissue genesis.

The use of biomaterials in wound healing can be traced back thousands of years to ancient times when cloth and poultice-like materials were applied to wounds. Similarly, compounds like honey, lint, and grease were used to aid in wound healing by Egyptians and other ancient civilizations [3]. Wound care has since then evolved over thousands of years to where we are today. Interestingly, the principles of dressing wounds to protect them and limit infection still hold true today. However, the birth of polymer chemistry in the last century and the coalescence of polymer scientists, engineers, and clinicians has brought about the development of newer wound dressings and therapies that continue to push the envelope of advancing wound care [4]. The goal of this review will be to provide a bridge between the clinical and material science perspectives of how wound dressings have been developed and the important characteristics of polymeric biomaterials to consider for designing the next generation of wound dressings.

## Overview of Wound Healing

Skin is the largest, yet often overlooked, vital organ of the human body. The role of the epidermal skin is to provide an external barrier from the outside elements, prevent desiccation or infection, and provide protection from mechanical, ultraviolet, and physical insults [5, 6]. Thus, a wound is defined as damage or disruption of the external epidermal barrier of skin that results in exposure of the deeper tissue structures to the outside elements and can result in significant morbidity if not closed efficiently and appropriately [5, 6]. Thankfully, our bodies have a natural physiological feedback loop, a process known as acute wound healing, that responds to injurious insults to the skin that works quickly to counteract and repair “open” wounds [7].

### Acute wound healing

**Fig. 2:**
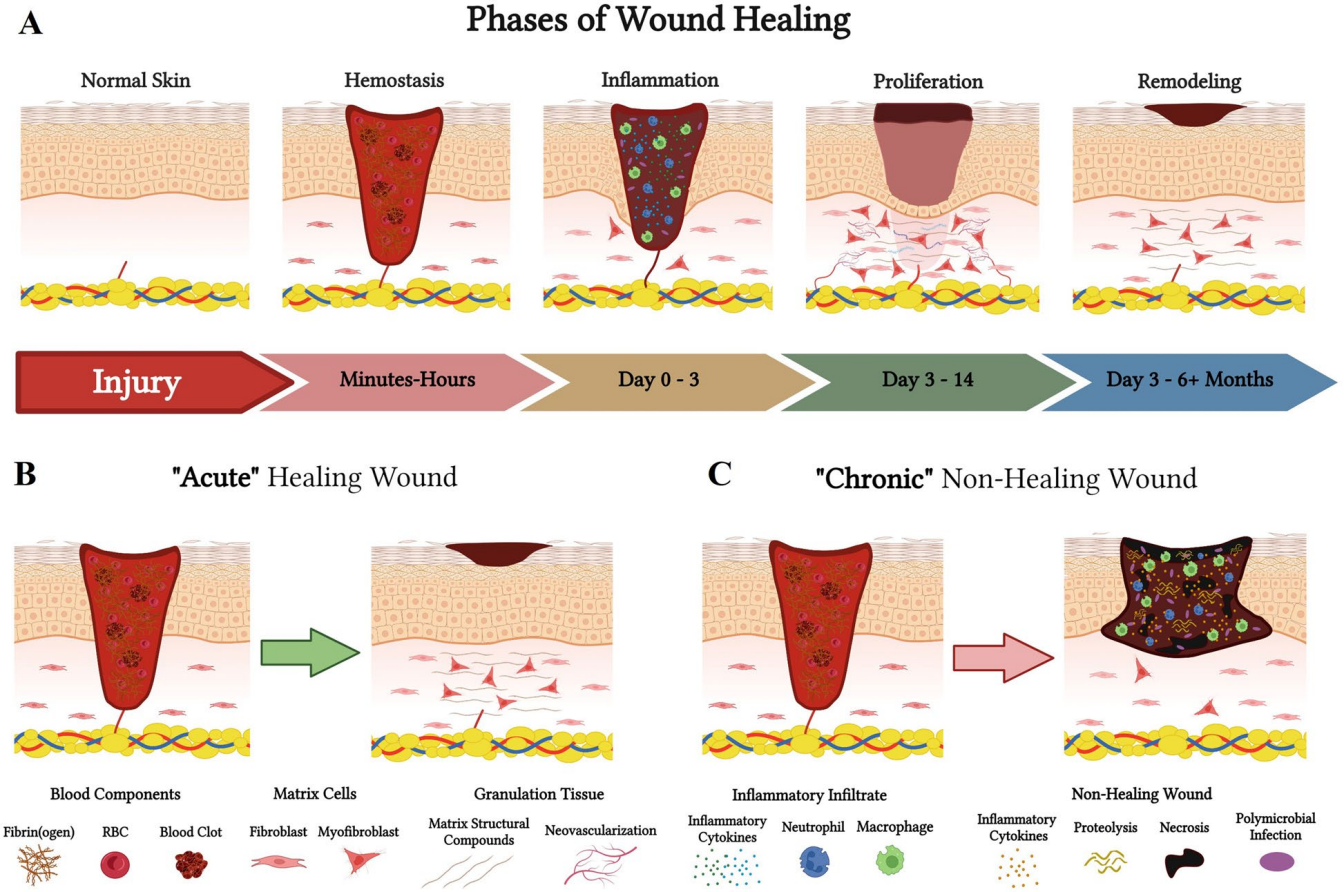
Phases of wound healing. Depiction of the phases of wound healing and comparison of acute versus chronic healing. **A** Progression through the physiological phases starting with uninjured skin progressing to remodeling and formation of a scab. Includes a time scale to compare temporality. **B** Depiction of recently injured wound in hemostatic phase of healing progressing to proper healing and scab formation. **C** Depiction of chronic wound not properly progressing from hemostatic phase through healing and scab formation resulting in ulcer formation and an open wound. Created using www.biorender.com software

Acute wound healing follows a physiological and dynamic signaling cascade upon injury that can be broken down into four component phases (Fig. 2A and B) [6]. Starting with the *hemostatic phase*, which is the immediate response of the local tissue vasculature to activate platelets and generate a clot via formation of a provisional fibrin-platelet matrix [8]. The goal of this phase is to prevent excessive blood loss and exsanguination, while also serving as an initiation signal for wound healing to commence [8]. Next, is the *inflammatory phase*, which is a series of immunomodulatory signaling cascades that results in immune cell migration (neutrophil and monocyte/macrophage) into the wound tissue to begin removing damaged debris, foreign objects, or bacteria [9]. The previously deposited fibrin-platelet clot serves as a biological signal as well as a temporary scaffold for invading cell populations into the wound site [10]. The inflammatory phase typically culminates in about one week. Subsequently, is the transition into the *proliferative phase*, which is the stage of neovascularization, re-epithelialization, fibroblast proliferation, and wound contraction [11, 12]. The key modulators of this phase of healing are fibroblasts and keratinocytes, and the main outcomes are formation of granulation tissue and a restored epidermal barrier, respectively [12, 13]. The final stage is the *remodeling phase*, which does not occur until the wound has been sufficiently closed via reestablishment of the external epidermal barrier. Fibroblasts are the main cells participating in the remodeling phase and are involved in both the deposition of new matrix and the enzymatic degradation of old matrix in order to ultimately restore a state of anatomical homeostasis and function [7, 14].

One cannot overstate the critical role of the extracellular matrix (ECM) in wound healing, consisting of a myriad of biophysical, biomechanical, and biochemical cues that orchestrate the wound healing process. Specifically, the instructive cues provided by the topographical architecture, biological factors such as growth factors anchored to the structural proteins of the fibrous matrix that are carefully regulated by protease and anti-protease activity, and adhesive binding sites that promote the migration and proliferation of cells within the wound site. Unfortunately, the hostile environment of many complex wounds can dysregulate these processes and results in non-healing wounds, which continue to be a problem clinically.

### Progression of chronic wounds

Prolonged or abnormal progression through the stages of wound healing results in pathological, chronic, non-healing wounds (Fig. 2C) [15]. There are a variety of factors that can promote the progression towards pathological healing, such as trauma (particularly recurring trauma), nutritional deficits, infection, surgery, chronic disease, and radiation [15]. Moreover, most chronic wounds tend to be stuck in a perpetual cycle of the inflammatory and proliferative phases of wound healing [16–18]. Ultimately, this leads to wounds that fall within the continuum of excessive scar tissue formation and fibrosis or insufficient scar tissue formation and ulceration [16–19]. Unfortunately, chronic wounds remain a significant burden on the healthcare system, affecting over 8 million people in the US at a cost of over $30 billion annually [20].

There are a variety of local and systemic factors that can have a detrimental impact on wound healing and subsequently result in non-healing wounds. Locally, chronic wounds tend to maintain a highly inflammatory, oxidative, alkaline, and proteolytic tissue environment, in addition to having a higher propensity for microbial colonization (especially biofilm) and infection, which ultimately results in obstruction of physiological healing [21, 22]. Notably, wound fluid from chronic wounds demonstrated the ability to rapidly degrade matrix structural proteins (e.g. collagen) and key signaling factors, further demonstrating the destructive capacity of the proteolytic imbalance of chronic wounds [23]. Systemically, comorbid conditions that are associated with inadequate supply of nutrients and waste transport (e.g. cardiovascular disease) and states of chronic inflammation (e.g. obesity and diabetes) contribute to non-healing wound progression [21]. Similarly, complex wounds that result in significant tissue involvement and destruction, such as those from extensive burns, traumas, or military-based incidents, are also highly prone to progression towards non-healing chronic wounds and require special attention [15].

All of the above mentioned factors are important to consider when generating a wound treatment plan for a patient who may have varying degrees of each. However, comorbid health conditions, such as diabetes, are considered to play one of the most significant roles in the development and progression of chronic, non-healing wounds, where up to 15% of diabetics develop ulcerative wounds with a greater than 50% recurrence rate [24]. Diabetic wounds inherently have an improper balance and composition of bioactive compounds within the tissue, resulting in inadequate neotissue formation [25]. Consequently, lack of wound closure results in polymicrobial infections, desiccation, and reinjury of the diabetic wounds, which remain the leading cause of non-traumatic lower limb amputations [26, 27]. Overall, chronic wounds are highly complex and variable, though they are often treated in a similar fashion with labor intensive and non-specific treatment modalities that can include continuous wound cleaning, debridement, surgery, antibiotics, oxygen therapy, and dressing changes [28]. Thus, developing more effective personalized wound therapies is a critical area of research.

### Physiological parameters within wound environments

Wounds are more likely to heal appropriately when they maintain a warm, moist, clean, and pH controlled environment, with open exposure of wounds to the ambient environment resulting in drying out and cooling of wounds, increasing the risk of infection and impeding overall healing [29, 30]. Maintaining a warm wound environment near native body temperature, between 35 – 38°C, has been shown to improve blood flow and delivery of immune cell populations to wound tissue, resulting in improved wound outcomes [31–33]. A wet or moist wound environment has demonstrated the ability to improve autolytic breakdown of dead tissue, promote angiogenesis, enhance the rate of re-epithelialization, and decrease scar formation [29, 30, 34]. However, the tradeoff is that permitting the wound to scab and dry provides protection from microbial colonization. Thus, careful antiseptic measures should also be considered when maintaining moist wounds. Healthy skin maintains a relatively neutral pH, whereas during acute physiological wound healing, wounds become progressively more acidic [35–37]. Notably, acidification of more alkaline chronic wounds has been shown to improve chronic wound outcomes [38, 39]. Lastly, infection of wounds drastically decreases wound closure and can result in progression towards a chronic wound [21, 27]. Therefore, any insults that prevent the tissue from achieving a warm, moist, clean, and pH controlled environment are likely to result in hindrance of physiological wound healing and deviation towards non-healing wounds. Interestingly, fetal wounds undergo a more efficient form of wound healing relative to post-natal wounds, often resulting in scarless healing [40]. Thus, the benefits seen post-natally by maintaining a warm, moist, clean and pH controlled environment likely, in part, recapitulate the conditions fetal wounds are exposed to in the womb.

Another important parameter is the relative oxygen abundance within the wound tissue. An initial state of transient hypoxia is considered a stimulus for wound healing through a HIF-1α-dependent mechanism that promotes enhanced stromal cell activity [41]. Conversely, a prolonged state of hypoxia inhibits wound healing and prevents adequate nutrient exchange for neotissue formation [42]. Chronic hypoxia is often a result of vascular insufficiency due to comorbid conditions, such as diabetes and peripheral vascular disease [24]. Whereas transient hypoxia is a result of disruption of local tissue vasculature upon injury, with the hypoxic environment acting as a signal to recruit inflammatory cells to the wound site, stimulate granulation tissue formation, and promote angiogenesis [42].

The relative abundance of reactive oxygen species (ROS) within many wound types is also important to consider when designing proper wound dressings. ROS are involved in the inflammatory processes of wound healing and include species such as superoxide anion ·O_2_^-^, peroxide ·O_2_^-2^, hydrogen peroxide H_2_O_2_, hydroxyl radicals ·OH and hydroxyl OH^−^ ions. ROS play a critical role in the antimicrobial oxidative burst activity of phagocytic cells and vascular activity (i.e. vasodilation and vasoconstriction) [43]. However, the prolonged inflammatory phase of chronic wounds can result in excessive ROS production and impairment of healing [43]. Thus, wound dressings that are prone to oxidative activity from ROS will be more labile within chronic wound tissue. If oxidation is important for proper dressing functionality (e.g. drug release via surface erosion), then this may be a desirable characteristic. Similarly, chronic wounds tend to maintain a relatively exudative, alkaline, and highly proteolytic profile [17, 24, 44]. Thus, polymeric dressings prone to enzymatic degradation (e.g. biopolymers like collagen) or hydrolytic degradation (e.g. synthetic poly (esters)) will be susceptible to being broken down and metabolized. Since most wound dressings are applied for a short duration of time, they are typically not prone to significant degradation. However, degradative kinetics can be a key parameter when designing polymer dressings for the purpose of serving as a drug delivery vehicle for controlled release of bioactive compounds that aid in modulating the wound healing environment [45].

## Goals of Wound Dressings

A pair of landmark studies by George Winter in the 1960s demonstrated that maintaining a moist environment enhances the rate of re-epithelialization, wound closure, and overall wound healing [29, 46]. This concept has become an essential pillar of wound care and is a major influencer on wound dressing design and methodology. Winter suggested that “composite dressings”, ones that included both hydrophilic and hydrophobic components, best achieved the desired goal of moist wound healing for enhanced epithelial migration, whereas progressive drying promotes epithelial maturation and hinders microbial colonization. To this day, there is yet to be a single class of materials most effective for all wounds, though occlusive or semi-occlusive dressings that create and maintain a moist environment are considered the foundation of wound care. As is discussed in this review, the ultimate goal of wound dressings are to serve as an adjuvant to augment biological wound healing. Thus, the appropriate progression through wound healing is not only dependent on the wound type and systemic factors, but also wound dressing design and material interactions within the wound site. Ultimately, wound dressings should be designed to aid in the progression of acute wound healing, prevent transition from acute-to-chronic wounds, help wounds undergo a chronic-to-acute reversion, or a combination of these processes.

**Fig. 3:**
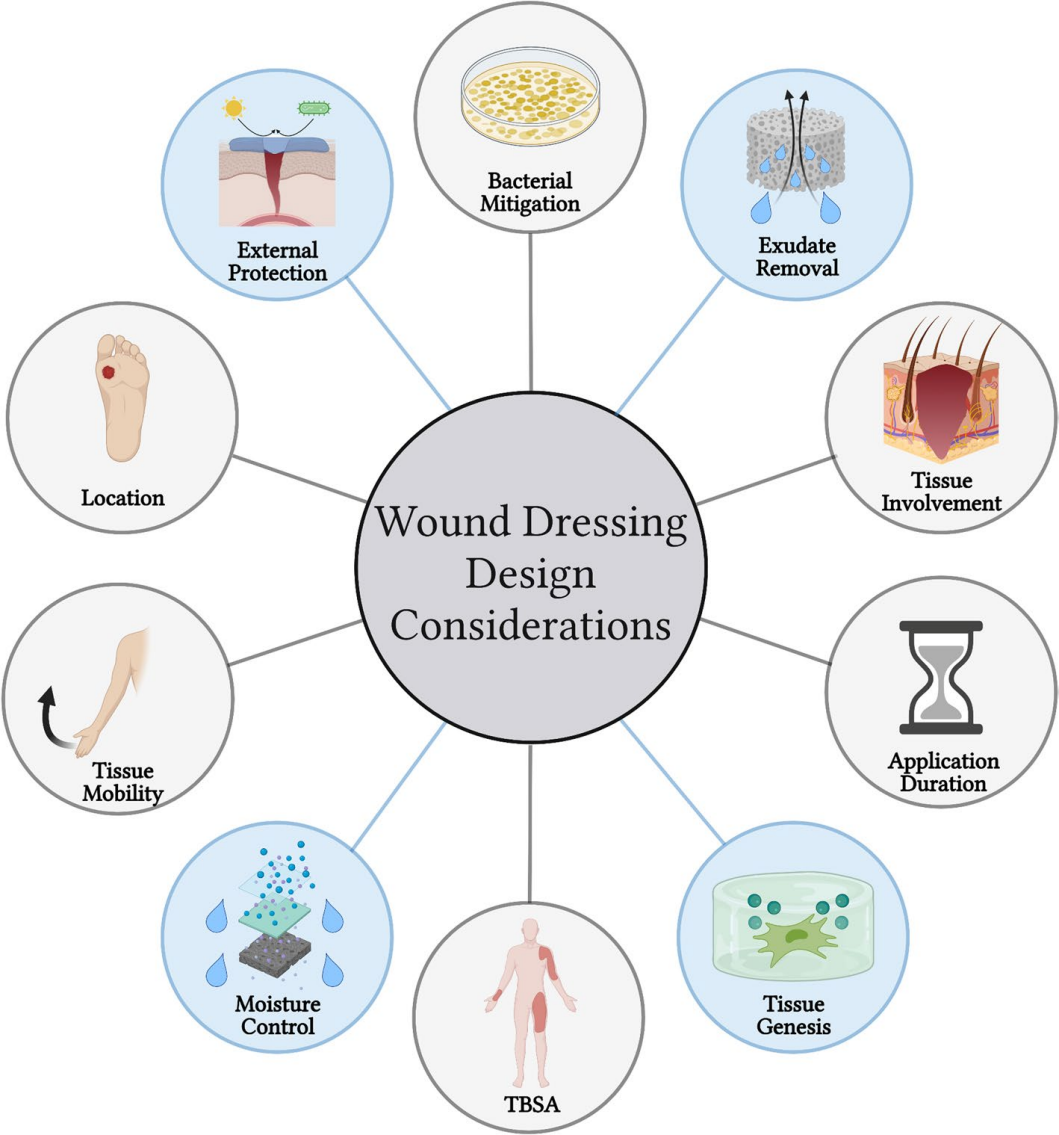
Web diagram of wound dressing design Considerations. Schematic diagram listing ten important characteristics to consider when design wound dressings. The four circles highlighted in blue represent the four design criteria listed within the texted as “key” parameters. The remaining six circles highlighted with grey are important supplementary parameters to also consider, although the degree of importance can vary depending on application. Created using www.biorender.com software

There are a variety of different ways and outcome measures used to classify desirable wound dressing characteristics. However, broadly speaking, there are four key properties that are important to consider when designing wound dressings: 1) Ability to cover exposed tissue and protect from external insults (e.g. UV irradiation, physical trauma, or infection), 2) aid in exudate removal, 3) prevent desiccation and maintain a moist environment, and 4) augment the tissue regeneration response to promote neotissue formation (Fig. 3). The fourth property is an important property to consider when developing wound dressings for more specific, tailored tissue responses. Other factors to consider as well are, location of the wounds on the body (e.g. flat surface vs. irregular contour), tissue types involved (e.g. fascia, muscle, bone, etc..), mobility of the tissue site (e.g. regular dynamic movement vs. immobilized), duration of application (e.g. permanent vs. transient), and extent of body surface area involved.

A common type of dressing used clinically for decades is the wet-to-dry gauze, which is simple and inexpensive but can be labor intensive and further damage the wound site if allowed to dry [17, 22]. Gauze is typically made of rayon, polyester, or cotton, which are varying forms of cellulose fibers derived from plants [47]. Other types of common wound dressings include plastic films, foams, alginates, hydrocolloids, hydrogels, and bioengineered dressings/grafts, all of which have a variety of formulations [48–52]. Thus, this review provides a generalized overview of different types of synthetic and biologically derived polymeric wound dressings, the pros and cons of each, how different wound applications benefit from different polymer properties, and the role they each play in different wound healing settings. Of note, many clinically utilized wound dressing modalities are proprietary in nature and thus the exact formulations are not public knowledge, though an attempt has been made to broadly characterize each category with respect to their material science background.

## Important Polymer Properties to Consider

Polymers are one of the most widely produced substances in the world and have become intimately involved in the facilitation of everyday life for humans, including the field of medicine. The term “polymer” is derived from the Greek words ***poly*** meaning “many” and ***meros*** meaning “parts”, and thus, polymers are often also called macromolecules because they consist of multiple repeating monomeric units (>10 repeat units) to generate large molecules (~10–10,000,000 Daltons) [53]. Polymers can be biological, synthetic, or semi-synthetic (modified biopolymers) and can consist of a single monomer repeat unit (i.e. homopolymers) or consist of more than one monomer repeat unit (i.e. co-/hetero-polymers). Ultimately, polymer science intertwines and connects the fields of chemistry, biology, physics, material science, engineering, and medicine in order to generate materials that cover a diverse range of mechanical, chemical, physical, and biological properties.

Polymers are typically broken down into three general classes, 1) Plastics, 2) Fibers, and 3) Elastomers. Plastics can then be further subdivided into thermoplastics and thermosets, and fibers can be classified as cellulosic or non-cellulosic. Polymerization reactions can be carried out a number of different ways, including condensation, free-radical, ionic, ring-opening, macromolecular substitution, group transfer, or enzymatically. After formation of desired polymers, post-processing can occur in order to further tailor polymer properties for specific applications, such as end-group methacrylation for forming crosslinkable hydrogels or covalent linkages of peptides/proteins [54–56]. Crosslinking of polymeric scaffolds is not always necessary but is typically required for hydrogels in order to improve mechanics and provide dimensional stability of the substrate. Crosslinking can be categorized as a physical (i.e. ionic, hydrogen, or hydrophobic interactions), chemical (e.g. Schiff base, thiol-ene, acrylate, or azide bonds) dependent crosslink and can be reversible or irreversible [57, 58]. Some of the most common ways to crosslink polymers include light (e.g. UV), thermal, physical, ionic, or enzymatic (e.g. thrombin) [59, 60]. Lastly, end application polymer-based materials, such as wound dressings, can be fabricated via a number of different manufacturing modalities, including injection molding, melt molding, extrusion, phase separation, woven or non-woven meshes, 3D printing, and electrospinning, all of which exhibit various levels of control over the structural and mechanical properties [61–64]. Thus, the ability to create a diverse range of characteristics of polymer-based materials with customizable properties is what makes polymers such an appealing option for developing wound dressings. This section will highlight a variety of important material science concepts and provide a general overview of how these polymer properties can be modulated to alter biomaterial functionality. The information in this section, though not exhaustive, will be important for understanding how to bioengineer and advance beyond the limitations of modern types of wound dressings discussed in the subsequent section.

### Molecular Weight

There are a number of different “molecular weight” values used to describe polymers, including the number-average (M_n_), weight-average (M_w_), and viscosity-average (M_v_) molecular weights. The molecular weight is a key property of polymers and can have significant effects on a variety of other polymer properties. Relatively speaking, increasing molecular weight of a polymer will increase its size, decrease its rate of degradation, modulate its mechanical properties, and alter absorptive capabilities. It is important to note that the synthesis of polymers results in a heterogenous distribution of polymer sizes, thus polymers are generally denoted with a range of molecular weights, denoted as dispersity (*Đ*) [65]. The extent of dispersity for polymer molecular weights often depends on the polymer class, composition of reaction mixture, and synthesis technique/conditions, which can have resonating effects on overall polymer characteristics and applications [65]. Dispersity is considered a crude parameter for evaluating polymer uniformity, and can be calculated with the equation of *Đ* = M_w_/M_n_. As *Đ* approaches a value of 1, the polymer is considered to approach monodispersity, though *Đ* = 1 is yet to ever be achieved in practice. However, with the recent advancement of techniques such as atom transfer radical polymerization (ATRP) [66], ionic polymerization [67, 68], nitroxide mediated polymerization (NMP) [69], and reversible addition–fragmentation chain-transfer (RAFT) polymerization [70], polymer synthesis has come close to a generating a homogenous, monodispersed population.

**Fig. 4:**
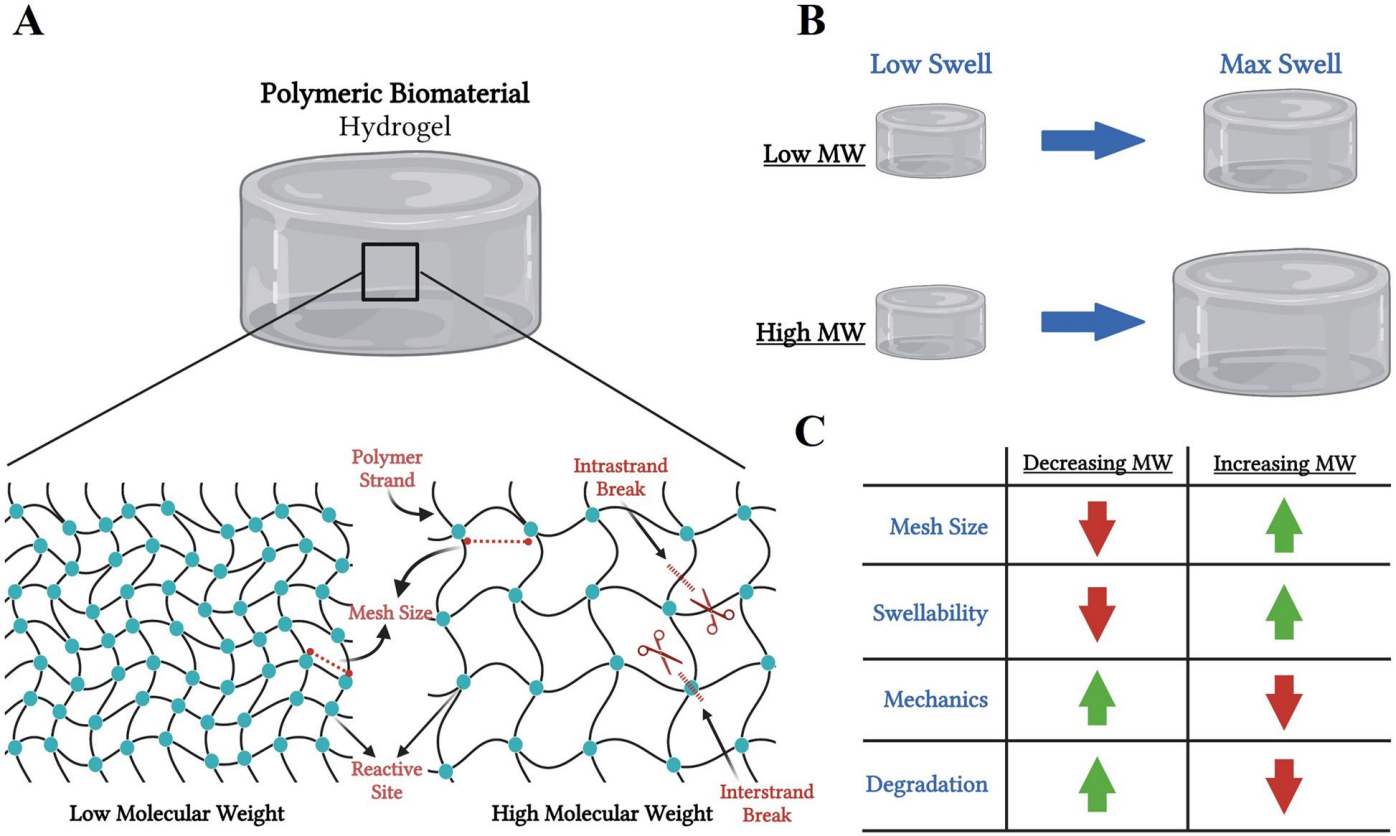
Polymeric hydrogel physical properties. **A** Depiction of a hydrogel model showing differences in mesh sizes between (*left*) low molecular weight polymers and (*right*) high molecular weight polymer hydrogels. The frequency in functional reactive sites can be seen and is depicted as teal circles at the junction point of polymer strands. **B** Schematic representing the function of molecular weight in the swelling of a polymeric hydrogel. **C** Chart of relative trends in polymeric hydrogels as a function of molecular weight. Created using www.biorender.com software

Molecular weight modulation is an especially important parameter when considering the design of wounds dressings, such as hydrogel-based dressings. Molecular weight can have significant effects on hydrogel network formation and overall mesh sizes, which is a common method to control the diffusional delivery rate of bioactive compounds (Fig. 4A-C) [71, 72]. Hydrogel mesh size is the linear distance between two adjacent polymer crosslink sites, thus increasing molecular weight increases the number of polymeric units and can decrease the frequency of functional crosslink sites by increasing the distance between crosslink units for most traditional hydrogel networks (Fig. 4A). Notably, frequency and activity of possible crosslink sites within polymeric units can be dependent on other properties as well, including polymer structure/chemistry, environmental conditions, and crosslinking methodology, therefore molecular weight is not the only parameter to consider.

In general, when keeping other parameters constant, increasing molecular weight will result in increased mesh sizes and swelling capabilities of a hydrogel, while also decreasing mechanical properties and the rate of degradation (Fig. 4B and C) [58, 72–74]. Conversely, decreasing molecular weight decreases mesh size and increases the mechanical properties of the hydrogel due to increased crosslinking per unit area, but decreasing molecular weight also often leads to a more rapidly degrading hydrogel due to increased frequency of the more reactive crosslinking sites and end groups, depending on the degradative mechanics (Fig. 4A and C) [58, 72–74]. Thus, depending on the context of wound type, specific polymer molecular weights can be applied to achieve desired effects, which is often important when utilizing hydrogels as temporary delivery vehicles versus long-term wound dressings [58]. Ultimately, molecular weight is only one of many parameters to consider, though it offers an easily controllable polymer property with predictable effects.

### Hydrophobicity

**Fig. 5:**
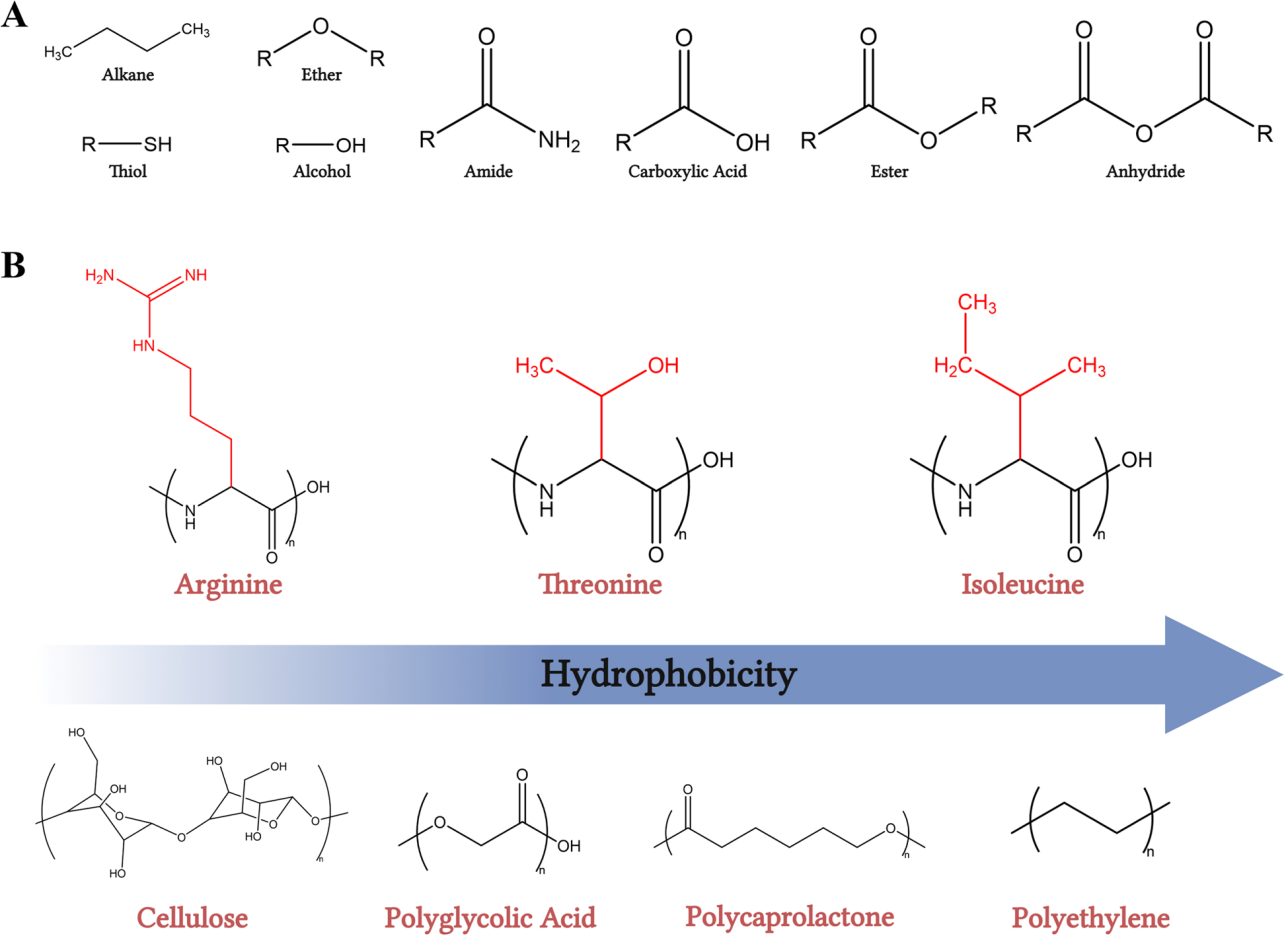
Material chemistry and hydrophobicity. **A** Depiction of the different functional groups that are commonly found in polymeric biomaterials and give rise to many of their properties. **B** Depicts a hydrophobicity scale with more hydrophobic (water-resistant) polymers including polymers with more hydrocarbons linkages and less hydrophobic polymers containing more reactive oxygen and nitrogen moieties. Includes different amino acids (*top*) and different synthetic monomers (*bottom*), in addition to cellulose (*bottom left*). Created using ChemDraw Office software

Hydrophobicity is the measure of reactivity a polymer has with water. Polymers that thermodynamically favor dissolution with water are labeled hydrophilic (water-loving) and polymers that favor dissolution in oils are labeled hydrophobic (water-resistant) (Fig. 5A and B) [75]. Hydrophobic polymers are structurally composed of long hydrocarbon chains (-H_2_C-CH_2_-) or contain aromatic rings (e.g. benzene) and thus, are more nonpolar in nature. Whereas hydrophilic polymers often consist of hydroxyl (-OH), carboxylic acid (-COOH), or amino (-NH_2_) functional groups and are more polar in nature (Fig. 5A and B). Hydrophobicity is important to consider when designing polymer-based biomedical devices, such as wound dressings. Polymers that are more hydrophobic in nature exhibit increased protein adsorption and moisture wicking properties [76], whereas hydrophilic components will increase absorptive capabilities of aqueous solutions. Depending on the context, protein adsorption may be advantageous or disadvantageous. However, it is believed that protein adsorption of blood proteins onto hydrophobic materials induces a unique morphological change in the protein structure due to hydrophobic interactions, which can expose protein epitopes that can promote the propagation of a foreign body response via inflammatory cell recognition [77–79]. Hydrophilic polymer dressings are typically seen in the setting of hydrogels and absorptive foams. Moreover, polar, hydrophilic polymers are more susceptible to hydrolytic breakdown within highly exudative wounds and thus can result in degradation and pH alterations within the wound tissue due to acidic byproducts [45]. However, acidification of chronic wounds has been shown to aid healing in many settings by increasing antimicrobial activity to mitigate bacterial burden and production of bacterial toxins, altering proteolytic activity, enhancing angiogenesis and tissue oxygenation, and improving epithelization [38, 45].

As previously discussed, maintaining a moist wound environment while simultaneously removing exudate are both key properties of wound dressings to consider. Many wound dressings are specifically designed to target this very principle. Absorptive wound dressings are hydrophilic dressings and have a continuous porous microarchitecture, which allows these polymer dressings to absorb and trap aqueous fluid [48, 49, 80]. The absorptive capabilities of some dressings can also be manipulated via modulation of the porous microarchitecture. Care must be taken to ensure removal of exudate is done while still maintaining a moist wound environment to avoid excessive drying out of wound tissue and inhibition of healing. Addition of a semi-permeable hydrophobic moisture wicking layer is often utilized to aid in exudate removal and can be incorporated as a base contact layer or superficial backing to a hydrophilic dressing. By combining hydrophobic and hydrophilic layers together within a wound dressing (i.e. Winter’s composite dressing), a wetting gradient can be created that allows for the absorption of exudate, while also permitting the regulation of moisture vapor transpiration [81–83]. Not all wound dressings are inherently comprised of both of these properties, but depending on the context of the wound type, a wound may not need both; for instance, healing of drier wounds can be hindered by absorptive dressings due to excessive drying, thus, a dressing that provides added moisture is more appropriate [50].

### Biodegradation/Bioerosion/Bioresorption

Polymers are broken down within the human body and degraded into smaller parts, known as biodegradation, bioerosion, or bioresorption depending on the context. We will use degradation as an umbrella term for the purposes of this review. Both larger polymeric structures, as well as the smaller degraded byproducts can ultimately interact with the body. The rate of degradation varies amongst polymers and depends on a variety of factors, including hydrophilicity, molecular weight, size, crystallinity, molecular structure, reactivity of labile groups, bonding, and environmental cues, just to name a few. Therefore, there is no single set value for how quickly polymeric compounds, such as a wound dressing, may degrade.

**Fig. 6:**
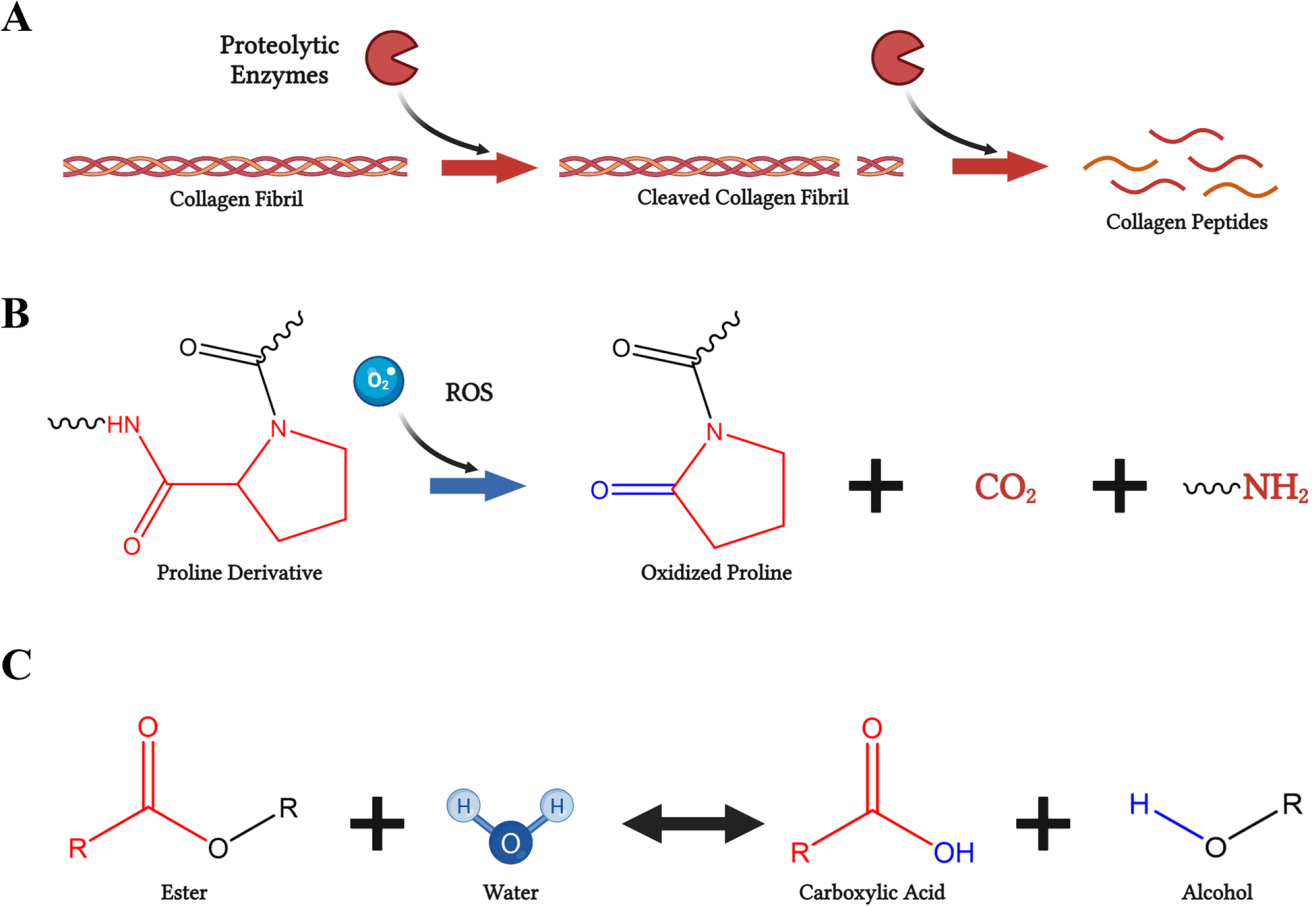
Polymer degradation mechanisms. **A** Enzymatic degradation depiction with proteolytic enzyme breaking down collagen fibril into smaller collagen peptides. **B** Oxidative degradation depiction with a reactive oxygen species degradation polymer with a proline derivative. **C** Hydrolytic degradation depiction of an ester-containing polymer reacting with water and broken down into an alcohol and carboxylic acid. Created using www.biorender.com software

**Fig. 7:**
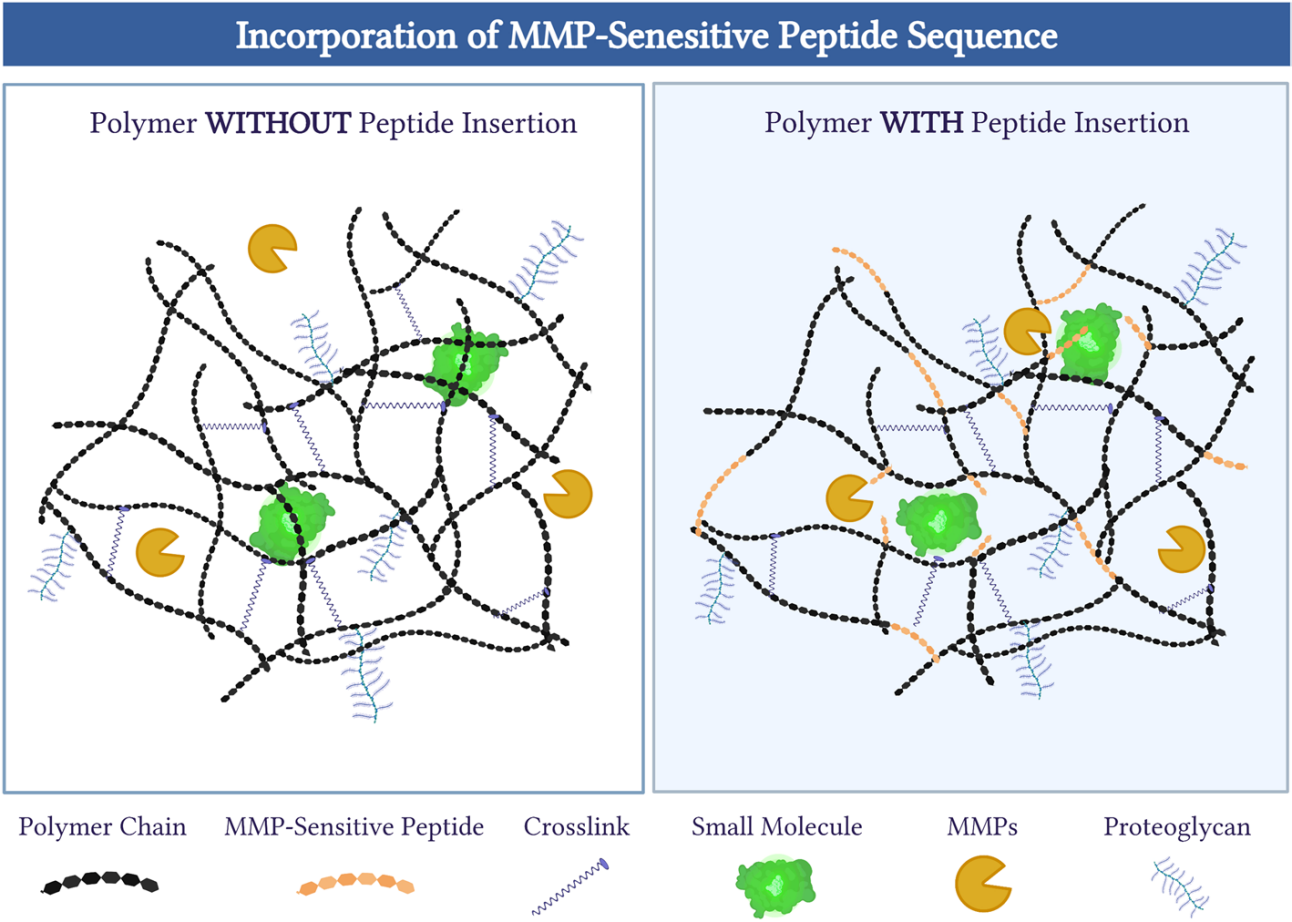
Insertion of peptide sequences into polymeric biomaterials. Diagram to depict how different peptide sequences can be incorporated into polymeric biomaterials to modulate their properties. Shown here is the insertion of an MMP-sensitive peptide sequence (orange polygons) that is inserted into individual polymer strands (black polygons) to allow for control over degradative kinetics and release of small molecules (green), such as drugs or biologics (*right*). Created and adapted from www.biorender.com software

**Fig. 8:**
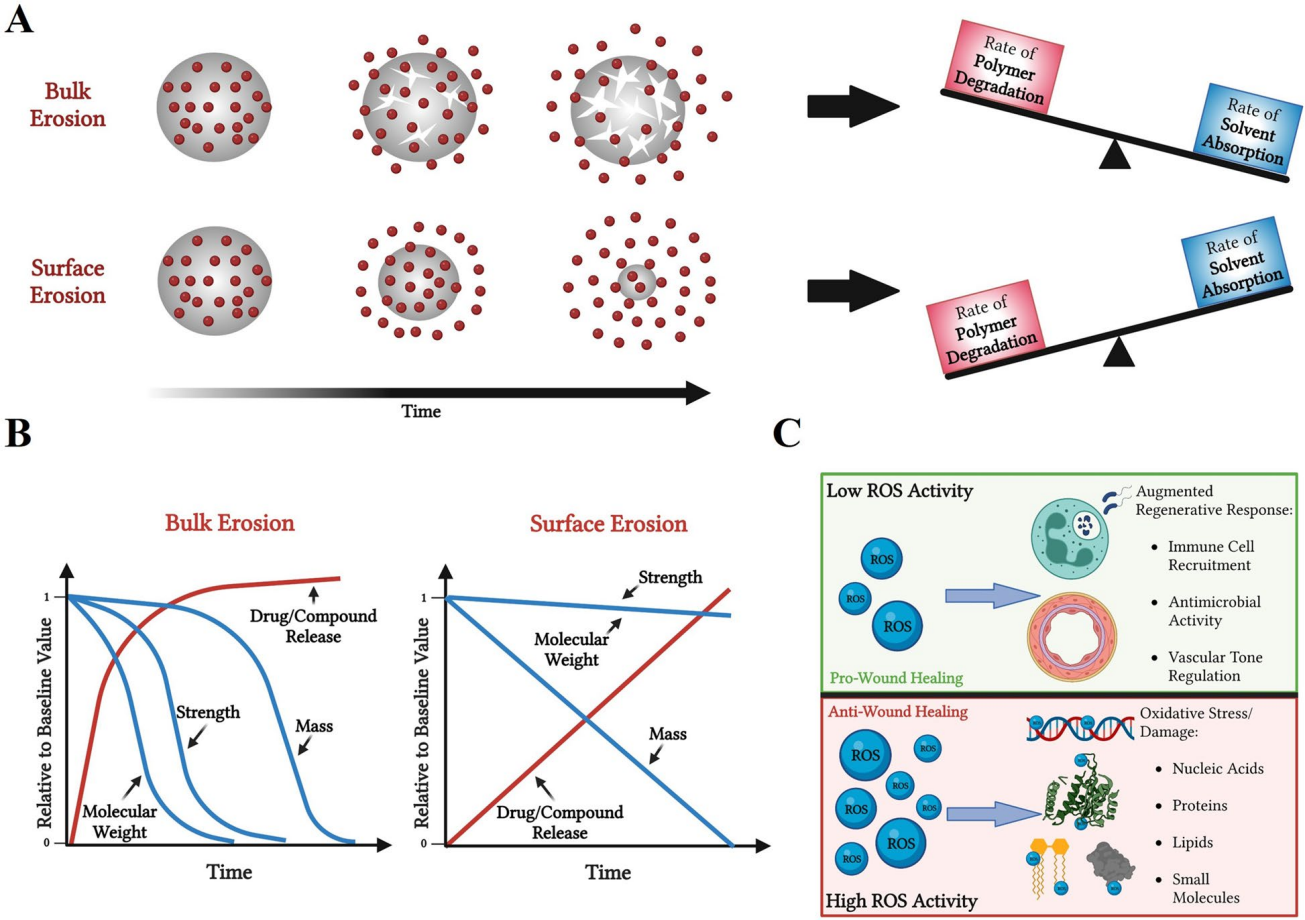
Bulk versus surface erosion dynamics. **A** Depiction of polymer structure (grey circle) that contains small molecules (red circles) within the polymeric structure. (Top) Demonstration of bulk erosion and more rapid burst release of small molecules due to the rate of solvent absorption being greater than polymer degradation, relatively. (Bottom) Demonstration of surface erosion and a more gradual controlled release of small molecules due to the rate of degradation being greater than solvent absorption, relatively. **B** Graphical representation of polymeric dressing properties and drug/small molecular release kinetics over time via Bulk (left) and Surface (right) erosion. Changes in polymer properties depicted in blue lines. Changes in drug release kinetics depicted by red line. **C** Schematic to represent the relative role of ROS compounds in wound healing. Created using www.biorender.com software

Polymers are degraded via one of three main mechanisms, enzymatically, hydrolytically, or oxidatively (the physical/mechanical disruption of bonds will not be discussed in the context of this review) (Fig. 6A-C) [84]. Most synthetic polymers do not degrade efficiently via enzymes which target specific amino acid sequences unless enzyme-sensitive moieties are introduced within the polymer network [58, 85]. However, peptide moieties are commonly incorporated into synthetic polymeric biomaterials to improve biocompatibility, cellular attachment, and degradation control, such as the incorporation of the amino peptide sequence *GPVGLIGK,* an MMP-2/9 sensitive peptide sequence (Fig. 7) [86]. Hydrolytically degradable polymers contain a higher relative composition of labile functional groups that react with water, commonly esters, anhydrides, acetals, carbonates, amides, urethanes and phosphates (Fig. 5A) [87]. Polymers that contain mostly hydrocarbons, such as polyethylene (PE) or polycaprolactone (PCL), are more hydrophobic in nature and do not tend to degrade as rapidly via hydrolysis. However, not all polymers with hydrolytically labile functional sites will significantly degrade in water; one must also consider relative hydrophobic properties, glass transition temperature (T_g_), and crystallinity of the polymer. Lastly, oxidative degradation of polymers occurs via reaction within ether-based bonds in the backbone and side chains of polymeric units. Oxidative degradation tends to occur via surface erosion with chain transfer of reactive oxygen species by water. Highly inflamed and chronic wounds typically contain abundant reactive oxygen species that can readily react with oxidative-sensitive moieties within polymer chains and other biological molecules (Fig. 8A-C).

Utilizing the degradation kinetics of surface erosion of a polymer is a popular technique for developing tailored time-release of drug compounds and can occur via both oxidative and hydrolytic degradation; whereas bulk erosion of polymers typically results in more rapid, burst release of compounds via hydrolysis (Fig. 8A) [45]. Degradation kinetics are important to consider when designing polymer-based wound dressings. For example, a hydrolytically sensitive wound dressing within a chronic wound environment may degrade faster, relative to acute wounds, due to the highly exudative and alkaline environment.

### Biocompatibility/Toxicity

Biomedical devices derived of polymers must maintain a high level of biocompatibility. Biocompatibility is often a broad and ambiguous term used to measure whether biomedical devices promote negative, unintended, or detrimental effects on tissue. However, it is not always accurate to consider biocompatibility of clinical interventions based on such qualifiers. For instance, the natural physiological immune response to a foreign dressing material over time is eventual encapsulation via the foreign body response. The foreign body response entails increased angiogenesis and granulation tissue formation, both key components of wound healing [88]. Thus, if a wound dressing prompts the foreign body response, such as that seen with Granufoam™ during negative pressure wound therapy (NPWT) [89], it could possibly be aiding in the wound healing response. In the right context and with proper control, it is reasonable to consider the trophic response of tissue after stimulation of the foreign body reaction to a biomaterial as desirable. Notably, the immunogenicity of biomaterial delivery vehicles has been shown to prime the immune system to generate a more robust response to vaccines [90–95]. Therefore, for the purposes of this review, we define biocompatibility to describe a polymer and its byproducts to be non-toxic and that they do not negatively impact the overall rate of wound healing and tissue formation, relative to if there was no clinical intervention. This definition focuses on the quantifiable result being rate of wound healing and tissue formation relative to no intervention, as opposed to whether the functional activity is deemed “beneficial” or “desirable”. Ultimately, it is important to consider the polymer, polymer byproducts, biomodulatory cargo, and direct physical interactions between the dressing and tissue.

### Mechanical Properties

**Fig. 9:**
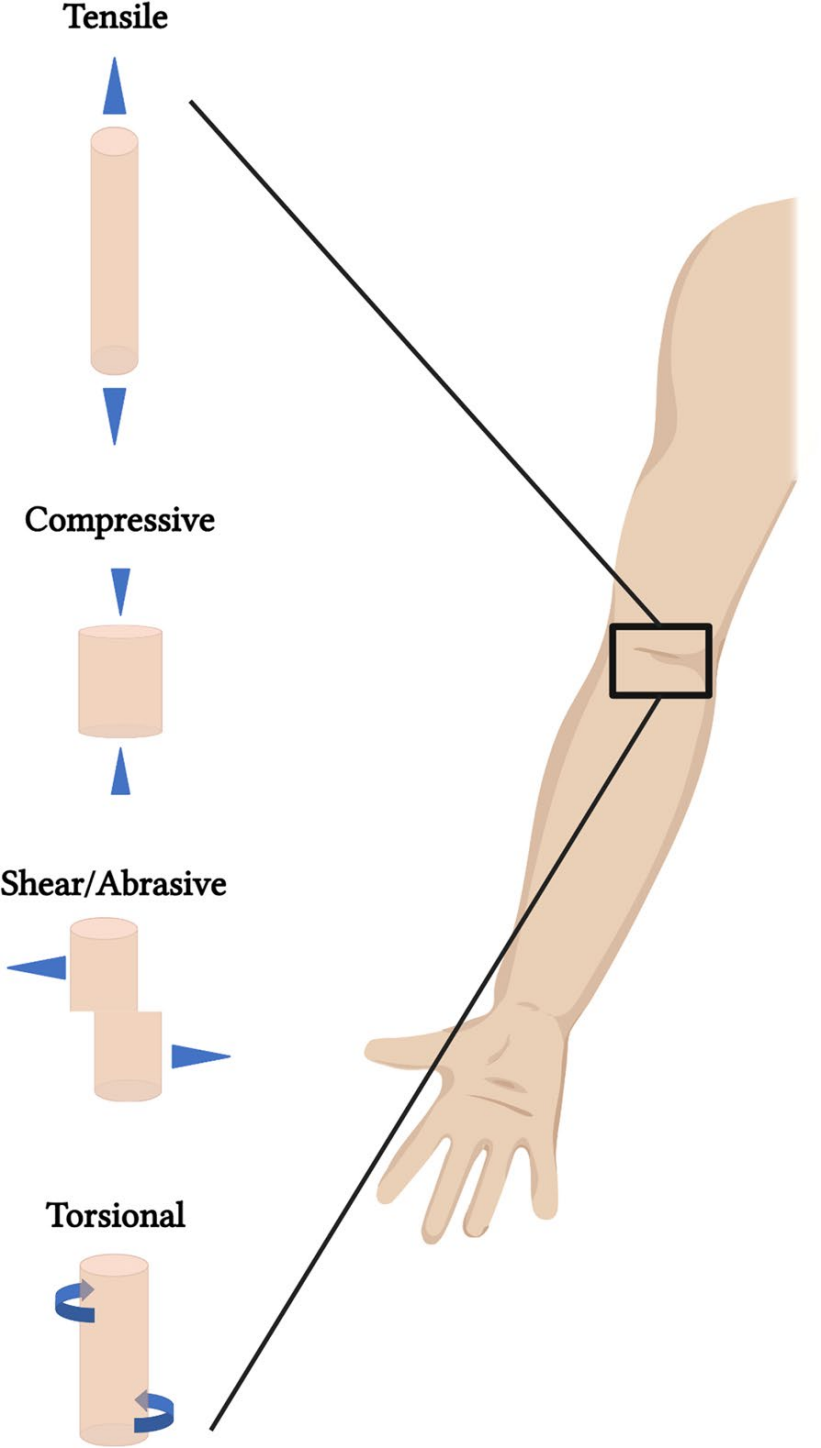
Native tissue force dynamics. Schematic representation of the common forces that skin tissue is exposed to. Created using www.biorender.com software

The mechanical properties of wound dressings are important at both the macro- and micro- level of tissue functionality and can be either static or dynamic in nature. Macroscopically, wound dressings provide support for the surrounding tissue and thus can be exposed to a variety of different mechanical insults depending on the wound type, location, and application it is being used for. Superficial wounds covered with films and bandages are typically elastic and flexible, especially for tissue locations that are involved in dynamic movements (e.g. elbow), but also tough enough to resist abrasive shear, torsional, and mild impact forces (Fig. 9). Non-superficial, complex wounds, often dressed with foams and hydrogels, may encounter additional environmental forces that require adequate compressive and tensile properties. For instance, an increasingly utilized wound healing modality is NPWT, which involves the insertion of a porous polyurethane foam into deep, complex wounds, and covering the foam with a secondary semi-permeable dressing that incorporates a vacuum source [96, 97]. Subsequent exposure to sub-atmospheric forces results in mechanical contraction of the wound site volume and has been shown to expedite the rate of wound closure in several wound types. Thus, the porous foam dressings used in NPWT must be flexible and compressible upon exposure to sub-atmospheric pressures, yet mechanically rigid enough to not be destroyed in such an environment. Moreover, complex wounds involving significant, support-tissue structures with high mechanical loads, such as muscle and/or bone, require more significant interventions. Current wound dressing modalities often fall short in providing significant mechanical support and dimensional stability while still being able to promote tissue regeneration in these types of high load-bearing tissues. However, more advanced tissue engineered wound dressings, such as custom 3D-printed scaffolds dosed with stem cells and/or regenerative biologics, are currently being explored and demonstrate promise in potentially improving outcomes seen in deeper, more complex wounds.

Microscopically, recent evidence suggests a significant role of wound dressing mechanical properties on overall wound healing signaling and outcomes via mechanotransductive signaling [98, 99]. Local cells residing within the tissue, including fibroblasts, epithelial, endothelial and progenitor cells, directly interact with the dressing material, but also indirectly respond to the physical disruption the dressing has on tissue mechanics. For example, the micro-deformations induced by porous foam dressing spicules on wound tissue has been thought to upregulate protein production and matrix production [98–103]. Thus, resident cells can “sense” and “adapt” to a variety of mechanical cues in the extracellular matrix and tissue environment during both physiological wound healing and upon interventional wound therapies [98, 99]. Review of recent literature further demonstrates the role of mechanotransductive, as well as topological, cues in augmenting fibroblast and stem cell maturation, function, and overall activity [104–108]. Notably, recent studies have revealed the potential critical role of tissue mechanics on epigenetic changes within cells, termed mechanoepigenetics, which is still in the early investigative stages [109–111]. Clinically, evidence has demonstrated how compression of wounds can be beneficial, though exposure to excessive compressive or tensile forces, such as over-suturing [112], can result in aberrant healing with fibrosis and hypertrophic scarring, possibly through induction of YAP/TAZ signaling [113].

### Permeability

**Fig. 10:**
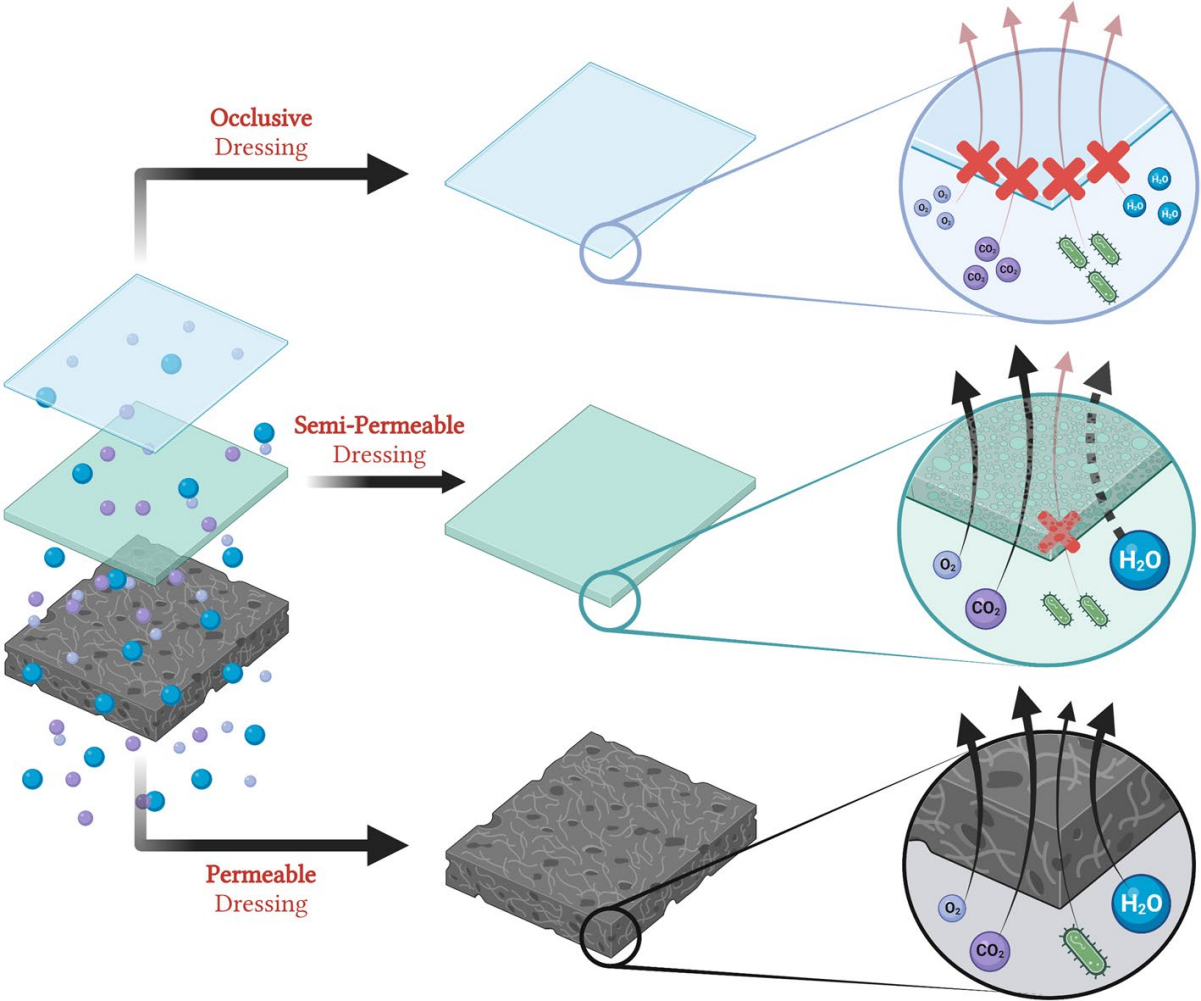
Comparing the relative permeability of dressings. Schematic representation of the different degrees of permeability a wound dressing contains. (Top, blue) Depiction of an occlusive or non-permeable dressing that is most commonly used as a superficial or outermost layer. Occlusive dressings prevent the movement of fluids, both gas and liquids, as well as cells and bacteria. (Middle, green) Depiction of a semi-permeable or semi-occlusive dressing that permits the movement of gases and water vapor (dashed black arrow) but typically limits the movement of liquids to variable degrees depending on the dressing. Semi-permeable dressings prevent the movement of cells and bacteria. (Bottom, black) Permeable or non-occlusive dressings are often depicted as foam or foam-like materials that are absorbent in nature and allow the movement of fluids, both gas and liquid, in addition to cells and bacteria. Oxygen molecules depicted as small blue circles. Carbon dioxide molecules depicted as small purple circles. Bacteria depicted as green organisms. Water is depicted as larger blue circles. Black arrows depict movement through the dressing material. Thicker arrows depict ability to evaporate into ambient environment. Red arrow accompanied by red “X” depicts lack of transport through material. Black-dashed arrow depicts that liquid water does not transport but water vapor still can. Created and adapted using www.biorender.com software

**Fig. 11:**
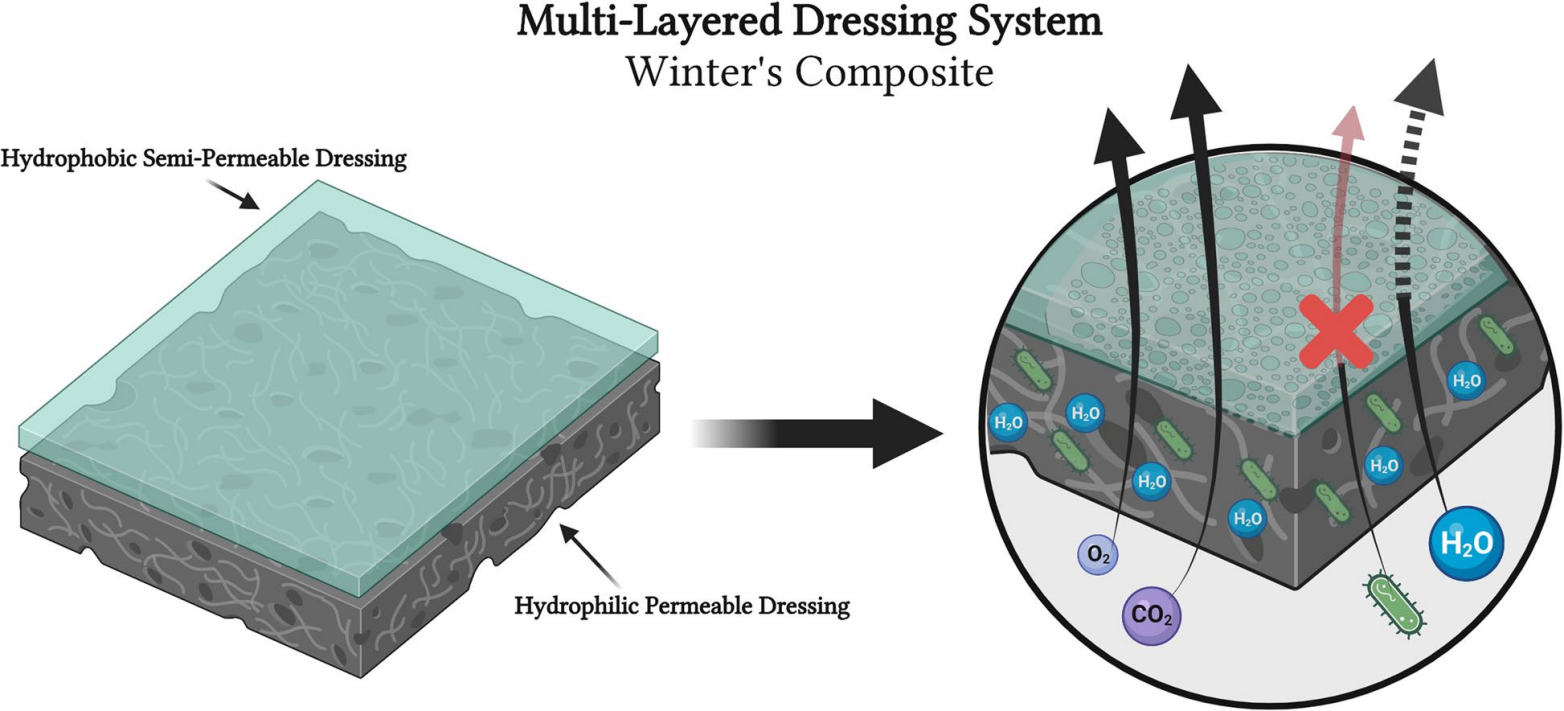
Example of multi-layered wound dressing system. Schematic representation of dual-layered wound dressing system, Winter’s Composite. Includes a hydrophilic, permeable base foam dressing layer (Bottom, black) covered by a hydrophobic, semi-permeable dressing layer (Top, green). Depicted in the composite dressing is the combined effects of a permeable and semi-permeable dressing, where all fluids and cells/bacteria can pass through the permeable foam base, but liquid water (and other liquids such as serous exudate) in addition to cells/bacteria get stuck within the permeable foam layer because they cannot pass through into the semi-permeable dressing on superficial surface. However, the semi-permeable layer still allows some removal of water through evaporation, where water vapor is allowed to pass but not liquid water. This combination, known as Winter’s composite, creates a permeability gradient and can aid in exudative removal in mildly exudative wounds, upon dressing changes, due to the absorptive hydrophilic foam. Oxygen molecule depicted as small blue circle. Carbon dioxide molecule depicted as small purple circle. Bacteria depicted as green organism. Water is depicted as larger blue circle. Black arrows depict movement through dressing material. Thicker arrows depict ability to evaporate into ambient environment. Red arrow accompanied by red “X” depicted lack of transport through material. Black-dashed arrow depicts that liquid water does not transport but water vapor still can. Created using www.biorender.com software

The permeability of a polymer wound dressing is another key parameter to consider [48, 51, 52]. A variety of different levels of permeability are utilized for specific wound applications (Fig. 10). Often highly permeable dressings that are non-occlusive are utilized for the removal of highly exudative wounds, such as through the use of absorbent gauze. Highly permeable dressings allow the movement of fluids, both liquids and gases, and even cells/bacteria [48]. Care must be taken to avoid infection in instances where non-occlusive, permeable dressings are used. Semi-permeable, or semi-occlusive, dressings are one of the more common formulations for most dressings [48]. These allow the flow and exchange of water vapor and gases, but not liquid or cells. Thus, these can help trap in moisture to aid in moisturizing the wound tissue. Notably, semi-permeable dressings are often capable of regulating moisture within the wound environment through a process known as moisture vapor transpiration [114–116]. However, in highly exudative wounds, addition of absorbent materials with frequent dressing changes are typically required to prevent maceration of wounds and excessive trapping of fluid [52]. Lastly, are the impermeable, or occlusive, dressings which tend to allow minimal gas and vapor exchange, though low levels can still occur [48]. Occlusive dressings should not be used in the setting of exudative wounds because they can also result in moisture trapping, maceration of periwound tissue, and an increased risk of infection [48]. Two approaches to generate a wound dressing with variable permeability properties are the use of a multi-layered system with a permeability gradient or the combination of multiple different wound dressing classes together (e.g. an absorptive foam covered with a semi-permeable film) (Fig. 11).

### Modifiability

Polymers are appealing for the generation of wound dressings, in part, because the already diverse profile of synthetic and natural polymers can be further modified in a distinct and controllable manner to generate tailored biomaterials [117–120]. In the field of tissue engineering, polymers are modified to alter their physical properties (degradation kinetics, mechanics, shape-forming ability, or microarchitecture), chemical interactions (ionic, acidic/alkaline, aqueous, or other bonding interactions), and biological activity (cellular interactions, promotion of tissue genesis, immunomodulation, and delivery of drugs/biologics). As our understanding of physiology continues to progress and polymer synthesis techniques become more advanced, the library of synthetic- and naturally- derived polymers continues to expand with enhanced versatility for fine-tuning polymer properties to modulate specific biological activity.

Perhaps one of the more transformational qualities in polymer chemistry is the versatility of functional group modulation within polymers [119–122]. End-chain, but also intra-chain, functional groups are common sites of chemical manipulation to alter the polymer macromolecular properties, including tissue-material interactions and controlled release of biologics and/or cells. Incorporation of selectively reactive functional groups into the polymer chain, such as hydroxyls, carboxylic acids, thiols, or primary and secondary amine containing compounds, are often utilized. For example, the chemical acrylation of polyethylene glycol (PEG) to form polyethylene glycol diacrylate (PEGDA) [123, 124]. Incorporating acrylate groups into the polymer chain (or acrylic acids to end groups) allows for physical or chemical crosslinking to improve mechanics, covalent binding of other polymers or bioactive compounds (e.g. growth factors), and incorporation of selective enzyme-sensitive sites for biologically controlled degradation.

As previously mentioned, altering the biological activity of polymer biomaterials is often desired. For example, by integrating naturally occurring extracellular matrix binding motifs, polymer properties such as material mechanics, cellular adhesion, and tissue integration can be controlled [125–128]. Similarly, proteins, antibodies, oligonucleotides and drugs can be covalently or non-covalently (adsorption, electrostatic interactions, hydrophobic interactions, or hydrogen bonding) affixed to polymer chains [129]. Cell-mediated release of covalently affixed compounds can be mediated via enzymatic cleavage of growth factors or other biologics that were previously conjugated to incorporated enzyme-labile moieties (MMP-sensitive) within the polymer chain. Conversely, non-covalent release is often a result of environmental or affinity-based mechanisms. Therefore, the benefits of controllable, diverse polymer compounds can be combined with distinct cellular bioactivity via selective modification of polymer chains and integration of bioactive compounds for dynamically responsive biomaterials. The potential ability to improve cellular delivery methodologies is important for the future development of next generation tissue engineered therapies. Altering the number of hydrolytically labile moieties, enzyme sensitive moieties, or controlling the crystallinity of the polymer, permits control over release kinetics of cells from polymer vehicles. However, one step further is the ability to control cellular phenotype, viability, and engraftment by modifying the material-cell interaction within the delivery vehicle, in addition to co-delivery of drugs and/or biologics with cells to achieve temporal-spatial control over cell fate.

### Synthetic vs Biologic

Not only are there synthetically derived polymers utilized for the fabrication of wound dressings, but there are also a number of biopolymer options that can be classified into the categories of polynucleotides, polysaccharides, or polypeptides (Fig. 1) [48, 49, 80, 130]. Notably, the most commonly used dressing in the world is gauze, which is derived of cellulose, a natural polysaccharide. Additionally, there are other plant and algae-based biopolymers, such as pectin, dextrin, and alginate, or chitosan which is derived from crustaceans, that are commonly used for wound dressings. Similarly, mammalian polysaccharides such as hyaluronan, chondroitin sulfate, and heparin are often used. Polypeptides are also garnering increasing interest in the field of tissue engineering. This is because the native tissue environment consisting of proteins, such as collagen, and polypeptides inherently contains a diverse range of physiological cues for tissue regenerative processes [131–133]. Thus, a variety of formulations are currently under investigation searching for ways to replicate the body’s natural biopolymer structure, in addition to its biomechanical and biophysical properties [134–137]. Polynucleotides are less commonly used for wound dressing applications but have been used for the purposes of providing a bioactive signal and thus, can be incorporated into wound dressings for the purpose of augmenting the tissue regenerative response [138, 139]. Alternatively, synthetic polymers consist of a diverse range of macromolecular compounds that typically allow for greater control of structure, chemistry, composition, and bioactivity, which ultimately allows for enhanced customization capabilities of synthetic polymer-based biomaterials. Technical approaches are commonly utilized to alter the synthetic polymer and/or the body’s immunological reaction to the synthetic polymer, to prevent undesirable immune responses and potential graft/dressing failure, such as incorporation of biomodulatory or anti-inflammatory compounds [140], such as TGF-β or IL-10.

## Examples of Traditional Wound Dressings

As previously discussed, the overarching goals of wound dressings are to protect the wounds, regulate moisture, and mitigate bacterial colonization in order to maintain a warm, moist, clean, and pH-controlled environment. Traditional modern dressings vary in their structure, function, mechanics, and bioactivity and include gauze, films, foams, alginates, hydrocolloids, and hydrogels. There are many advantages to each respective dressing class depending on the clinical context, with modern dressings significantly improving wound care and patient outcomes (Table 1). However, there are limitations to modern wound dressings, often due to the broad “one size fits all” approach in their designs.

**Table 1:** Characteristics of common polymeric dressings

Dressing Class	*Polymer Component(s)*	*Natural or Synthetic*	*Bacteria/Infection Control*^*a,b, c*^	*Permeability*^*d*^	*Exudative/Moisture Control*	*Wound Types*	*Limitations*^*e*^	*Commercial Products*
**Loose-Weave Gauze (Dry/Wet)**	Cellulose-derived	Natural	**• Dry:** Outside-In - External barrier protection dependent on thickness and fiber weave **• Wet:** Inside-Out - Bacterial removal dependent on external absorptive gradient	Non-Occlusive; Fiber weave and thickness can alter permeability	• Absorptive; aids in removal of exudate. Wetness, coating, and fiber weave alter absorption. **• Wet-to-Dry:** A technique where premoistened gauze creates an Inside-Out moisture gradient (frequent dressing changes).	• Moderate-to-Highly exudative wounds. Wound protection as a secondary dressing in many wound types.	**• Dry:** Reinjury of tissues upon removal if enmeshing occurs. Requires dressing change when exudate absorption "strikes through" to outer surface. **• Wet-to-Dry:** Requires dressing change every few hours to maintain moisture gradient or is prone to bacterial overgrowth.	Surgical Gauze and Kerlix®
**Fine-Mesh Gauze (Coated/ Uncoated)**	Cellulose-derived	Natural	Inside-Out - Dependent on secondary dressing for external absorptive gradient.	Semi-permeable; Tight fiber weave (traditional 44/36; warp/weft) reduces permeability.	**• Uncoated**: Absorptive, although single ply is thin without much capacity, requiring secondary dressing which must then be changed. • **Coated:** Maintains moisture when coated with hydrophobic ointments, preventing drying and reducing scabbing.	• Wounds that are shallow and superficial. **• **Often used for promoting re-epithelialization, of which coated fine-mesh gauze is often ideal.	**• Uncoated**: Requires secondary dressing. Can become incorporated in scab. **• Coated:** Still requires secondary dressing but maintains moisture and reduces scab. Decreased absorptive properties.	Scarlet Red®, Xeroform®, Vaseline Gauze
**Films**	*Polyurethane*, *Silicon*, Nylon, Polyester, or Polyethylene	Synthetic	Outside-In - External barrier protection	Semi-Permeable or Occlusive	Non-absorptive; traps moisture within wounds. Permits moisture vapor transpiration.	• Dry-to-Minimally exudative wounds. • Secondary external dressing to hold other dressings.	• Moisture trapping can result in tissue maceration and bacterial overgrowth. • Minimal mechanical protection. • Pain upon removal due to adhesion.	Tegaderm®, Covaclear®, Opsite®, Darmatac®
**Foams**	*Polyurethane, Silicone*, and Polyvinyl Alcohol	Synthetic	Variable - Dependent on pore size and hydrophobicity	Variable (typically Permeable-to-Semi-Permeable)	Variable absorptivity; dependent on hydrophobic moisture gradient. PVA will dry to rigidity if exposed to air.	• Can be used on most wound types, though typically utilized with Moderate-to-Highly exudative wounds. • Composite dressing are common for foams.	• Can trap bacteria within the foam dressing. • Can dry out some wounds. • More rigid foam dressings can induce tissue injury at a micron level. Reinjury of tissue upon removal due to enmeshing. • Requires wound monitoring and dressing changes every 1-7 days.	Allevyn®, Granufoam®, Optifoam®, Aquacel®, Bioatain®, Polymem®, Hydrofera Blue®
**Alginates**	Mannuronic and Guluronic Acid	Natural	Inside-Out - Dependent on inherent antimicrobial properties	Permeable or Semi-Permeable	Highly Absorbent; aids in removal of exudate and moisture from wounds.	• Moderate-to-Highly exudative wounds. • Can be used to promote re-epithelialization.	• Can cause excessive drying of wounds. • Often require secondary dressing to hold in place.	Kaltostat®, Algisite®, Sorbsan®, Seasorb®
**Hydrocolloids**	Silicone or Polyurethane backing. Carboxymethylcellulose (CMC), pectin, or gelatin colloid layer	Natural or Synthetic	Outside-In - External barrier protection	Semi-Permeable or Occlusive	Moderate Absorption; can remove wound exudate while also donating moisture to wounds via colloidal gel.	• Dry-to-Minimally exudative wounds.	• Colloidal gel can breakdown in wounds via hydrolytic and enzymatic activity. • Requires monitoring of wound and dressing changes every 5-10 days.	Replicare®, Duoderm®, Exuderm®, Comfeel®
**Hydrogels**	Variable (Cellulose, Alginate, Polyethylene Glycol, Polymethacrylate)	Natural or Synthetic	Minimal - Dependent on secondary dressings and added antimicrobials	Permeable or Semi-Permeable	Minimal absorption; though provide moisture to wounds.	• Dry-to-Moderately exudative wounds. • Complex and deeper wounds.	• Can over saturate wounds and cause maceration. • Prone to hydrolytic and enzymatic degradation in exudative wounds. • Typically must be paired with additional dressings due to minimal mechanical or adhesive properties.	Amerigel®, Aquaflo®, Aquaform®, Intrasite®, Nu-gel®

^a^Outside-In: Prevents infiltration of exogenous bacteria

^b^Inside-Out: Helps eliminate bacteria residing within wound

^c^All dressing classes can be modified to incorporate in antimicrobial compounds

^d^Pore size is inversely related to permeability of gas, liquid, bacteria, and tissue

^e^Tissue enmeshing is dependent on pore size and permeability of dressings

### Gauze

As previously mentioned, gauze is one of the most commonly utilized and fundamental wound dressings in the world. Gauze is derived from cellulose, the most abundant polymer on the planet. Cellulose is a natural, homopolysaccharide, linear polymer capable of forming both crystalline and amorphous structures and is derived from glucose monomer units linked through β-1,4-glycosidic bonds [141]. Notably, cellulose can theoretically be degraded via oxidation, hydrolysis, or enzymatically via glucosidases/cellulases (bacterially-derived) [142–144], though the three-dimensional structure and hardy crystalline nature makes it exceedingly difficult to breakdown within the context of a wound bed. Additionally, the degraded byproducts are non-toxic glucose moieties. Traditional gauze comes in either woven or non-woven forms, is highly absorbent and permeable, is commonly utilized for exudative wounds, and typically acts as a non-occlusive dressing so it is prone to increased rates of bacterial colonization without proper management [52, 145]. Gauze does not naturally hydrate tissue or modulate bioactive signaling within the wound. More recent formulations involve impregnating fine-mesh gauze via addition of water, oil, or other bioactive compounds, such as antibacterial silver derivatives [52, 146]. Impregnated gauze is less absorbent and is typically not suitable for highly exudative wounds [52]. Gauze dressings most commonly are associated with a secondary dressing material to aid in holding the gauze in place and help modulate moisture level within the wound, such as with films, wraps, or adhesive tapes of differing porosity and permeability. Moreover, antimicrobial impregnated fine-mesh gauze formulations have garnered interest due to their ability to reduce the rate of infection, leading to improved wound outcomes [48, 147].

Although wet-to-dry gauze dressings remain one of the leading wound dressings used clinically, there are a number of limitations to this modality [148]. As previously mentioned, gauze often must be wetted first in order to prevent the wound from drying out; dried gauze within a wound can result in impaired healing [148]. Impregnating fine-mesh gauze with hydrophobic coatings, such as petroleum jelly (Vaseline), has been utilized in order to improve the moisture retention properties [4]. Moreover, more traditional loose-weave gauze dressings are prone to integration with granulation tissue and tissue enmeshing if left in the wound for extended periods of time and results in disruption of healing tissue within the wound upon removal of the dressing [149]. Thus, modulation of gauze fiber count and sizes has been used as a way to control tissue enmeshing while maintaining absorptive capabilities [4]. A gauze sheet is referred to as fine-mesh gauze when its pore sizes are small enough (typically measured as a fiber warp/weave of 44/36) to resist enmeshing of granulation tissue and encourages re-epithelialization beneath it. Additionally, the benefits seen with incorporating antimicrobial compounds into gauze dressings suggests that controlled release of impregnated biomodulatory compounds within gauze dressings could offer a potential avenue for regulating the inflammatory and trophic responses within wound tissue. Similarly, future incorporation of smart devices/sensors into gauzes dressings (and others dressings as well), permits the continuous and easy inpatient or outpatient observation of wound characteristics, such as exudate production and bacterial infiltrations to better denote when to change a dressing or administer antimicrobial therapy.

### Films

Adhesive film dressings are thin, flexible dressings that are often transparent in nature and are commonly used as a secondary dressing to seal the wound. The permeability and transparency of film dressings are often dependent on a variety of factors, including polymer chemistry, crystallinity, or T_g_, in addition to manufacturing modality and environmental stimuli. Adhesive film dressings are commonly derived of polyurethane, silicone, nylon, polyester or polyethylene materials often with a hypoallergenic acrylic adhesive layer around the edges, and are considered semi-permeable; allowing water vapor, oxygen, and carbon dioxide to pass through but preventing water and bacteria from passing [48]. Semi-permeable films are capable of providing a moist environment while also removing small amounts of liquid via a process known as moisture vapor transpiration (measured by moisture vapor transmission rate; MVTR). Adhesive films when used alone do not have a high enough MVTR to prevent excessive moisture trapping in moderate-to-highly exudative wounds. Thus, due to the moisture trapping and inability to regulate large fluid volumes, semi-permeable films are able to maintain a moist wound environment conducive of autolytic debridement, but due to moisture accumulation are also more prone to promoting wound/periwound maceration and bacterial overgrowth [48].

Overall, semi-permeable adhesive film dressings are typically considered to be comfortable relative to other dressings, and the transparent characteristics allows for constant wound observation [52]. Additionally, the thin elastic film can offer some protection of wounds from mechanical shear forces, while still permitting flexibility in dynamic tissues, but pale in comparison to the protective effects of other dressing modalities [52]. Films are typically reserved for dry, superficial wounds and should be avoided in infected, deep, heavily exudative wounds or in the setting of patients with fragile skin, if possible [52]. Adhesive films can be paired with an absorbent dressing layer, such as a foam dressing, for more exudative wounds, but still require regular dressing changes. Additionally, the use of films, particularly with the addition of silicone to increase sealing and skin comfort, can be used with adjuncts such as intermittent irrigation and/or the use of mechanical or motorized vacuums to increase air flow and thus reduce and control moisture vapor content. Ultimately, thin wound films are often limited in their capacity to act as a single dressing modality and are often consider a supplementary dressing or part of a multi-layered, composite dressing system. However, fabricating thin adhesive films capable of both drug delivery and wound monitoring, while permitting greater moisture control could bolster the efficacy of film dressings.

### Foams

Foam dressings are a diverse class of wound dressings that have been utilized in a variety of settings due to their wide ranging compositions and porous nature, including thin foam dressings for topical applications and bulky, porous foam dressings for insertion into deeper, full-thickness wounds [150]. The ability to modulate the main polymeric component (typically polyurethane or silicone) and a variety of different structural components, give rise to a versatile wound dressing class [150]. Commonly, microarchitectural properties are modulated to alter the overall macrostructural characteristics, including alteration of size, morphology, distribution and composition of the pores. Ultimately, the benefits of foam dressings can be stratified into four key categories, physical, mechanical, chemical, and biological [150]. Physical benefits of foam dressings include moisture control, various degrees of permeability, thermal insulation, and absorptive properties. As with any wound dressing, there must be a balance between absorptive properties and moisture maintenance, and this can be controlled via stratification of hydrophilic and hydrophobic polymeric layers, in addition to the stratification of variable pores sizes. Mechanical benefits include the often more rigid/stiff nature of foam dressings for deeper wounds, which provide greater protection from mechanical and/or physical insults, relative to other types of wound dressings, such as films. Though, the highly porous nature of most foam dressings provides a flexible and compressible substrate, with some exceptions. Similarly, foam struts and spicules can inflict micro-deformation and micro-strain on wound bed tissue, which has been shown to increase the production of matrix proteins important for wound healing [99–101]. Chemical benefits include the wettability of foam dressings, incorporation of adhesive components, and ability to deliver drugs in a controlled environment. However, the absorptive nature of most current foam dressing formulations makes controlled drug delivery more difficult. Lastly, are the biological benefits of foam dressings, such as the protection from microorganisms, prevention of tissue necrosis, delivery of bioactive compounds, and subsequent improvement in wound outcomes. Notably, due to larger pores sizes and common use in deep, non-superficial wounds, some foam dressings are prone to tissue ingrowth and enmeshing of neotissue which can provoke pain and reinjury upon removal [151]. Thus, foam dressings are often changed anywhere from every 1-7 days depending on the formulation, application and wound type.

Foam dressings are utilized for exudative wounds that are both superficial and non-superficial (i.e. deep to the epidermis), often times complex in nature, such as traumatic or chronic ulcerative wounds [48, 80, 150]. The diverse characteristics and versatility of foam dressings allows for their application in a variety of wound settings. For instance, many foams are polyurethane-derived, and due to the diverse nature of polyurethanes, the foams can be both hydrophilic and hydrophobic [152]. Aside from the popular use of polyurethane-based foams, are silicone-based foams that tend to be softer and more malleable which allows greater moldability to the irregular shape/contour of the tissue [48, 150]. Moldability is an important feature of silicone-based foams because more efficient surface contact can allow for enhanced mechanical and moisture wicking properties, often associated with increased rates of wound closure. However, current clinically utilized foam dressing formulations are restricted by their pre-formed nature which limits their tailorability to specific wound shapes and contours. Therefore, future formulations looking at injectable, pourable, and sprayable foams that expand and/or cure *in situ* provide additional personalization of foam dressings for the complex and variable nature of wound care, including the ability to treat deeper wounds via endovascular or percutaneous modalities. Additionally, designing foams with dynamic pore gradients with the capacity to tailor moisture control and drug/biologics delivery to wounds would drastically expand the breadth and efficacy of foam-based dressings.

### Alginates

Alginate is a naturally occurring ionic biopolymer isolated from brown seaweed and used to fabricate a variety of different wound dressing formulation, including hydrogels, films, and foams [153, 154]. Alginate dressings are sodium and calcium salts comprised of mannuronic and guluronic acid units, and thus are nontoxic and considered biocompatible in nature [49, 153, 154]. Alginate is found to be naturally diverse with wide ranging ratios of guluronate-to-mannuronate residues (G:M) [153, 154]. Thus, alginate inherently maintains a diverse profile of physical properties and molecular weights based on the relative composition of G and M residues [49]. Alginate typically forms a block of G and M residues either in a consecutive pattern (GGGGGMMMMM) or an alternating pattern (GMGMGM) [153]. Alginate gels are formulated with either acidic precipitation or ionic crosslinking with cations (Ca^2+^) [153]. Additional processing of alginate gels via freeze-drying can result in the formulation of foams and fibrous sheets [153]. Notably, ions within alginate-based dressings can promote the generation of a protective film upon exposure to blood proteins and maintain bioactivity beneficial to wound healing in some circumstances [155].

Biodegradable alginate wound dressings are highly absorbent, and thus are used in the setting of exudative wounds [49, 153]. Additionally, alginate dressings have been shown to intrinsically have antimicrobial, hemostatic, and immunomodulatory properties [153]. However, the absorptive properties paired with the permeable nature of alginate dressings can result in excessive drying of wounds and are not recommended for dry wounds. Therefore, alginate dressings often require a secondary dressing to superimpose over the top of the alginate dressing to prevent excessive drying of the wound. Additionally, alginate dressings may invoke an allergic response in some individuals and are non-adherent, thus requiring a secondary dressing. The ability to formulate alginate as a film, foam, mesh, or gel offers a unique opportunity to take advantage of the naturally absorbent, hemostatic, antimicrobial, and immunomodulatory properties of alginate in a variety of settings. Creating hybrid polymeric dressings composed of alginate would likely provide immense benefits to current dressing formulations for a variety of applications.

### Hydrocolloids

Hydrocolloid dressings typically consist of a two-layer wound dressing system, an inner colloidal layer and outer semi-occlusive/occlusive layer [52]. The inner colloidal layer is often self-adhesive and contains hydrophilic, gel-forming polymer compounds, such as carboxymethylcellulose (CMC), pectin, or gelatin [52]. The inner colloidal layer will form a protective gel cushion over the wound upon contact/absorption of wound exudate due to the hydrophilic nature of the colloidal polymer particles [52]. The outer layer is typically a semi-permeable foam or film, often a formulation of polyurethane, that is permeable to water vapor but impermeable to bacteria [156]. The outer layer seals the wound and can help protect from additional damage via external insults [156].

Hydrocolloid dressings are ideal for low-to-moderately exudative wounds and are effective in providing a moist wound environment that promotes granulation tissue formation and re-epithelialization, while preventing infection and not requiring a secondary dressing [52, 156]. Colloidal dressings are often used for superficial and partial thickness wounds and can remain on wounds for longer periods of time, upwards of 7-10 days, though care must be taken to monitor whether the dressing becomes saturated with exudate [156]. Evidence has demonstrated the benefit of hydrocolloid dressings over traditional gauze dressing in the treatment of chronic wounds [156]. However, the inner gel layer can begin to breakdown within the wound environment and should be monitored [157]. Ultimately, the benefits seen with hydrocolloid dressings warrants further investigations into future iterations that are fabricated with a colloidal layer that permits greater control over the degradation and possible delivery of bioactive compounds. Moreover, generating a more stable and transparent hydrocolloid would offer the opportunity to continuously monitor the status of the wound tissue and potentially extend the time in-between dressing changes.

### Hydrogels

A new age of hydrogel dressings have emerged, garnering renewed interest due to their chemical, structural, and biological diversity. Hydrogels consist of a hydrophilic polymer that forms a crosslinked network polymer and can absorb up to 1000x its dry weight in water, thus ultimately able to achieve >90% water content [52, 158, 159]. Different crosslinking techniques are used to form hydrogels, including the physical crosslinking methods of ionic bonding, hydrogen bonding, and hydrophobic interactions, in addition to the chemical crosslinking methods of free radical polymerization, conjugation chemistry, and enzymatic modalities [160–162]. Hydrogels are produced via a variety of different fabrication techniques including sheets, films, fibers, foams, nanoparticles, and coatings and can be injected, sprayed, or spread into a wound. Hydrogel dressings are able to provide moisture to the wound site, though addition of a secondary dressing is often desired to limit drying out of the hydrogel and to protect the hydrogel dressing, which often have limited mechanical properties. Counterintuitively, hydrogels tend to absorb minimal exudate because they are already saturated with water, however, changes in osmolarity and degradation can lead to changes in swelling within the hydrogel after application [158]. Additionally, hydrogels are non-occlusive and permeable to water and gas exchange. However, the moist environment and network structure permit bacterial colonization, limiting their use as an independent dressing without careful antiseptic measures [52]. Moreover, the low absorptive capacity of hydrogels lends to over-wetting of wounds and possible maceration if not properly addressed.

As mentioned above, hydrogels are a diverse and growing class of wound dressings. Both synthetic and biologically derived polymer formulations have been investigated. More recently, hydrogels have been investigated as a potential delivery vehicle for antimicrobials, biologics, drugs, and cells. The diverse class of polymer chemistries, ability to embed molecules, and ease of modulating the microarchitecture of hydrogels offers a conducive environment for controlling the delivery kinetics of bioactive factors capable of augmenting the wound environment [163, 164]. Encapsulation of compounds and cells within a secondary polymer vehicle within the hydrogel is another commonly utilized approach in tissue engineering [164, 165]. Thus, the molecular weight and degradation kinetics of the hydrogel polymer delivery vehicles are important for determining the release kinetics of the drugs and/or cells to the local tissue [165]. Additionally, the environmental factors within the wound site including hydrolytic, oxidative, and proteolytic activity, are critical factors to consider that can modulate the polymer properties and release kinetics, depending on how the bioactive factors are anchored within the hydrogels.

The tailorability and capacity to augment the tissue response with bioactive factors and cells is what makes hydrogels a desirable approach to chronic, non-healing wounds [163–165]. In this specific environment, there is a sustained state of inadequate growth factor bioavailability, overriding inflammation, and excessive proteolysis. Thus, delivery of counteracting factors in a temporal-spatial manner allows for the ability to potentially reverse this process in more difficult to treat wounds and improve wound outcomes not seen in traditional wound dressing modalities. Therefore, hydrogels are considered one of the more enticing bioengineering approaches for developing advanced wound therapies.

## Recent Advancements in Polymeric-derived Dressings

### Bioengineered Skin Substitutes

**Fig. 12:**
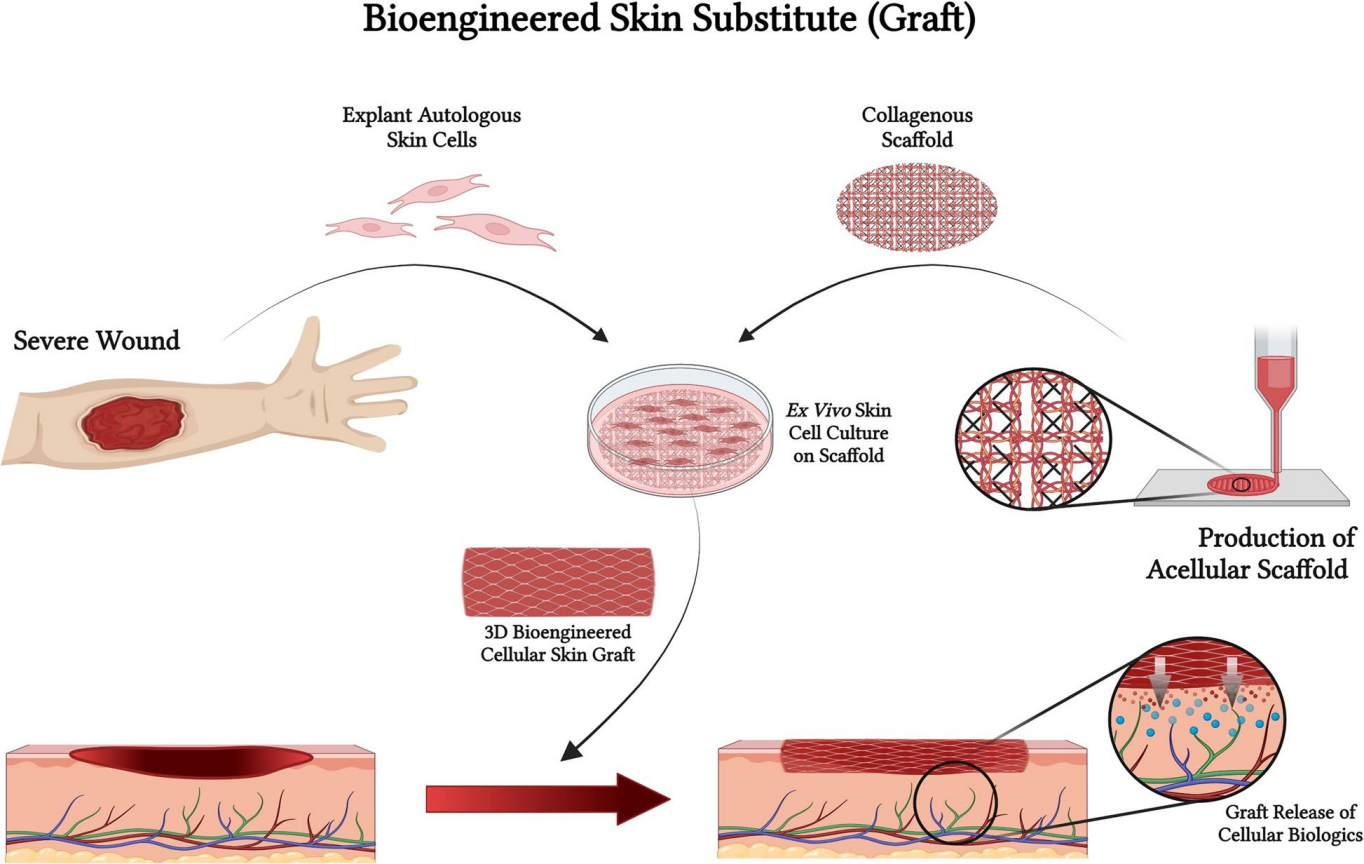
Fabricating a Bioengineered Skin Substitute (Graft). Schematic representation of generating a skin graft with autologous skin cells (i.e. keratinocytes and/or fibroblasts). A biopsy of a patient can be performed to remove autologous skin cells which can then be culture onto/within a polymeric scaffold *in vitro*. The scaffold can be fabricated a number of ways, depicted here is the methodology of 3D printing of a collagenous lattice. The skin cells are cultured on the polymeric scaffold for typically several weeks and then removed from cultured, and can be applied to a patient as a customized, autologous skin graft using their own cells. The graft is thought to work via a number of mechanisms, including coverage and protection of the wound, the embedded skin cells secrete biologics to promote wound healing within the native tissue, and the graft matrix can serve as a healthy tissue substrate for resident wound cells to grow onto/into and repopulate

More recently, bioengineered cellular-based living skin substitutes (grafts) have emerged and are currently still in the earlier stages of clinical use, though their use has grown tremendously in the last several years (Table 2). Relative to more traditional wound dressings and skin grafting techniques, they have shown mixed results in some clinical contexts but overall have demonstrated promise and offer a pathway for advancing wound care [166]. Currently, bioengineered skin grafts are considered to be non-inferior to autologous skin grafting and often superior to traditional wound dressing modalities in many clinical situations [167–173]. They consist of a range of synthetic, semi-synthetic, and biologically-derived polymers and polypeptides, often with a cellular component that consists of autologous or allogeneic human fibroblasts and/or keratinocytes (Table 2) [166, 167]. Most bioengineered grafts that utilize biological polymers (collagen) are derived from animal sources, such as porcine or bovine (Fig. 12).

**Table 2:** Common polymeric-derived skin substitutes

Product Name	Composition		Classification	
	*Polymer Component(s)*	*Cellular Component(s)*	*Clinical Use(s)*	*Note(s)*
**Dermagraf®**	Polyglactin Mesh	Allogeneic Neonatal Fibroblasts (*Human*)	Burns, Skin Grafting, Chronic Wounds	Improvements in wound healing time and graft success, shown to be less painful and easier to remove than allografts
**Apligraf®**	Bilayered Type I Collagen (*Bovine*)	Allogeneic Keratinocytes (*Human*) and Allogeneic Neonatal Fibroblasts (*Human*)	Burns, Skin Grafting, Chronic Wounds, Diabetic Ulcers, Epidermolysis Bullosa	Bilayered living skin equivalent, promotes cellular/tissue ingrowth
**Orcel®**	Bilayered Sponge of Type I Collagen (*Bovine*)	Superficial Allogeneic Keratinocyte Layer (*Human*) and Deep Allogeneic Fibroblast Layer (*Human*)	Chronic Wound, Skin Grafting	Bilayered living cellular matrix, often used as an overlay dressing on skin grafts to improve function and cosmesis
**Hyalomatrix®**	Bilayered Hyaluronan Scaffold, Outer Silastic Membrane	Autologous Fibroblasts (*Human*)	Burns, Chronic Wounds	Delivery of hyaluronan to wound bed, with a silicone membrane that acts as a temporary epidermal barrier
**Stratagraft®**	Bilayered Type I Collagen (Murine)	Superficial Allogeneic Keratinocyte Layer (Human) and Deep Allogeneic Fibroblast Layer (Human)	Burns, Skin Grafting, Chronic Wounds, Diabetic Ulcers,	Bilayered living skin equivalent, promotes cellular/tissue ingrowth
**Biobrane®**	Inner Nylon Mesh, Outer Silastic Membrane, Polypeptide Coating (*Porcine*)	N/A	Burns, Skin Grafting, Chronic Wounds	Shown to be particularly beneficial for pediatric populations
**Integra®**	Collagen (*Bovine*), Chondroitin-6-Sulphate (*Bovine*), Outer Silastic Membrane	N/A	Burns, Skin Grafting, Chronic Wounds, Other Soft Tissue Defects	Outer silastic membrane can peel away as dermis heals. Superior healing time relative to auto-/allo-/xeno-grafts
**Matriderm®**	Matrix of Type I Collagen (*Bovine*) and Elastin (*Bovine*)	N/A	Burns, Skin Grafting, Chronic Wounds	Matrix is structurally intact and promotes cellular/tissue ingrowth

Bioengineered skin grafts can be used temporarily to promote tissue growth, prime a skin grafting site, or serve as a more long-term replacement of tissue [166, 167]. Currently, bioengineered skin grafts are typically reserved for more complex wounds, such as partial or full thickness wounds, that involve more extensive damage to deeper anatomical tissues, but can be used for superficial wounds as well [166, 167]. Of note, not discussed in detail in this review, is the use of autologous skin grafts for the treatment of a variety of complex wounds, such as burns. Autologous skin grafts are often considered the standard of care for many complex partial and full thickness wounds, such as burns, and have demonstrated benefit over traditional wound dressings [174]. However, autologous grafting requires removal of skin tissue at specific locations from a donor site on the patient resulting in subsequent injury of that site. Additionally, patients with extensive wounds covering large portions of the body, such as with severe burns, make finding adequate graft sites not feasible. More recent, is the practice of *ex vivo* culture expansion of autologous skin cells or skin tissue to re-implant back into patients (Fig. 12).

One presumed mechanism of bioengineered grafts is that they provide a basement membrane-like layer for new epithelial growth due to the natural matrix-derived polypeptides structures (e.g. collagen, laminin, fibronectin), while the grafted cells also serve as a vehicle to deliver growth factors via cellular secretion before they ultimately undergo apoptosis. Consequently, the growth factors from grafted cells can induce migration of the native autologous epithelial cells. Thus, the premise of bioengineered skin grafts is that they more closely resemble native tissue, include biological substrates, and often contain cells capable of secreting a heterogenous milieu of growth factors, which ultimately expedites the wound healing process to a greater extent than traditional wound dressings (Fig. 12). Whereas more traditional wound dressings, previously discussed in this review, work by attempting to promote a warm, moist, clean, and pH balanced wound environment, and historically do not typically resemble native skin tissue.

Similar to the above method of fabricating bioengineered biological scaffolds from *ex vivo* expansion of autologous cells, is the recent investigations into decellularized tissue grafts [175]. Tissue can be isolated from a variety of sources, including human amnion, bovine tissue, and fish [176–178]. The process of decellularization removes the unwanted cellular components of the tissue to decrease the risk of rejection and failure of engraftment [179]. Decellularized animal/human tissue is biologically derived and thus, contain many of the important extracellular matrix compounds, such as proteins like collagen, that aid in the tissue regeneration process. Many decellularization processes and tissue sources are currently under investigation in clinical trials for a variety of different wound types, but often are utilized in more severe cases such as burns, traumatic, and chronic wounds. Ongoing studies are looking to improve the decellularization process to enhance the retainment of native biochemical and structural tissue properties, which can often be disrupted during the process of decellularization.

### Modifying Traditional Wound Dressings

**Fig. 13:**
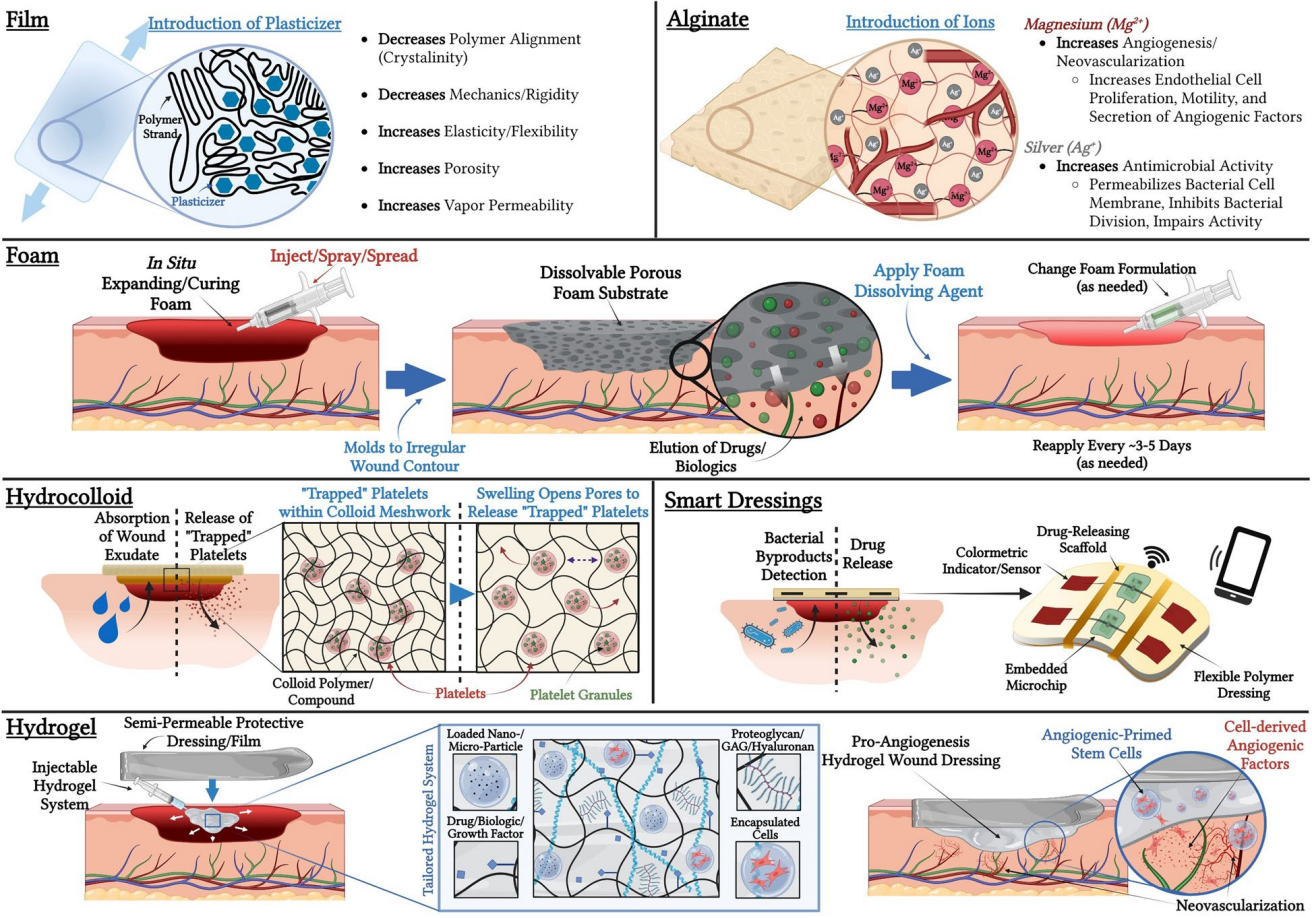
Modifying traditional wound dressings (Graft). Schematic depictions of ways that current traditional wounds dressings have been modified to enhance their wound healing capabilities. (*Film*) Insertion of a plasticizing agent, such as glucose or other small molecules into a polymer network can prevent the alignment of polymer fibers and subsequently increasing the flexibility of film dressings. (*Alginate*) A number of ions have been investigated for wound healing capabilities, such as the use of magnesium to enhance angiogenic signaling via modulation of native endothelial cells, and silver as an antimicrobial agent that has been used for decades. (*Foam*) Depiction of an *in situ* curing/crosslinking foam that expands to fill the irregular contour of many wounds to increase surface contact area. Additionally, foams can be embedded with drugs and/or biologics that can subsequently be released into the wound bed to promote controlled wound regeneration. (*Hydrocolloid*) Recent investigations in hydrocolloids have shown how drugs, biologics, and platelets can be delivered into the wound bed. Platelets have been investigated as a rich source of growth factors and immunomodulatory compounds via degranulation of their intracellular cargo. Release of platelets can be controlled a number of ways, shown here is how absorption of wound exudate results in swelling of the colloidal network and subsequent release of platelets. (*Smart Dressing*) Smart dressings can, in theory, be incorporated into a number of different dressing types via insertion of a small, flexible electronics. Depicted here a bacterial compound sensing smart dressing that allows for real-time monitoring of wounds, such as burns, ulcers, or surgical, for bacterial infiltration. Upon detection a sensor can provide both a visual color change in the dressing, in addition to sending a signal to a phone app for outpatient monitoring, and a drug-eluting scaffold can then be triggered to release antimicrobial compounds. (*Hydrogel*) Schematic depiction of a hydrogel formulated to be deposited into a wound and then a secondary semi-permeable dressing can be applied superficially to protect the hydrogel. The hydrogel can be dosed with a number of bioactive compounds and cells, such as the use of angiogenic-primed stem cells. The angiogenic-primed stem cells demonstrate enhanced angiogenic activity within the wound and release compounds that promote neovascularization within the wound tissue

Traditional wound dressings often fall short when it comes to treating chronic, non-healing wounds, which remain very difficult to treat due to their heterogenous and complex nature (Table 3) [180–207]. Thus, future wound dressing design looks to improve upon the limitations previously highlighted in current traditional wound dressing formulations, by not only improving their current properties that help promote a conducive healing environment but taking the next step in tailoring their antimicrobial and biological activity (Fig. 13). Additionally, several studies have begun to demonstrate that many of the traditional modern wound dressing classes are more efficacious upon merging into a hybrid dressing instead of a stand-alone dressing.

**Table 3:** Overview and description of common wound types^a^

Wound Type	*Depth of Tissue Involvement*	*Wound Description*	*Common Location(s) of Wounds*	*Common Insufficiencies*	*Most Common Dressing(s) Utilized*	*Design Considerations*	*References*
**Diabetic Ulcer**	Epidermis, Dermis, Subcutaneous, Fascia, bones and joints	Often of neuropathic etiology. Progression of simple acute wound towards chronic wound due to vascular and nerves damage. Inadequate blood/oxygen/nutrients supply and waste removal.	Plantar aspect of foot, tip of toe, lateral or fifth metatarsal, lateral malleolus and other pressure points	• Vascular • Neurological (Somatic) • Epithelialization • Matrix Deposition • Bacteria and Biofilm Infection	• Saline soaked gauze or impregnated gauze with silver or other antimicrobial. • Foams, and hydrogels that aid in bacterial mitigation. • Non-adherent dressings most common.	Off-weighting neuropathic pressure points. Targeting the promotion of re-epithelialization and angiogenesis, while regulating inflammation and matrix deposition are key factors. Hydrogel-based dressings for the controlled delivery of drugs, growth factors, stem cells and immunomodulatory factors have shown promise. Needs vary with depth of penetration.	[28, 180–183, 208]
**Pressure Ulcer**	Epidermis, Dermis, Subcutaneous, Fascia	A localized chronic wound induced by chronic contact pressure to the skin or soft tissue site. Occurs at the site of a bony prominence or location of compression from a medical device, frequently in insensate area or with depressed level of consciousness.	Sites of bony prominences (e.g. heel, ischium, trochanter)	• Muscle Mass • Skin Atrophy • Epithelialization • Matrix Deposition • Bacteria and Biofilm Infection	• Saline soaked gauze. • Foams, hydrocolloids, and hydrogels that aid in bacterial mitigation and moisture control. • Often associated with a polyurethane or silicone film or superficial cover. • Negative pressure wound therapy often employed.	Targeting the promotion of re-epithelialization and matrix deposition with neotissue formation are key design factors. Offloading needs and depth of wound will also affect dressing design. Hydrogel and fibrous polymer based dressings for the controlled delivery of stem cells and stem cell derived byproducts appear promising.	[183–188]
**Vascular Insufficiency Ulcer**	Epidermis, Dermis, Subcutaneous, Fascia	**Arterial insufficiency**: Terminal digit necrosis. Marginating skin loss with poor quality subcutaneous base distal foreleg. **Venous Insufficiency:** Similar appearance distal foreleg but hemosiderin staining present.	**Arterial:** Terminal digits, distal foreleg. **Venous:** Distal foreleg	• Vascular • Epithelialization • Matrix Deposition • Bacteria and Biofilm Contamination • Exudate Control	• Alginate dressings or other absorptive dressings with hydrating features such as hydrocolloids and foams. • Incorporation of antimicrobial therapy is common. • Compression and elevation of leg is important in venous insufficiency	Targeting the promotion of angiogenesis and neotissue formation are key design factors as is the inclusion of compression in venous insufficiency. Biomaterial-based dressings and nanoparticle for the controlled delivery of pro-angiogenic factors, such as VEGF and FGF, have demonstrated benefit. Stem cell based therapies are also promising.	[28, 185, 189, 190]
**Burn**	Epidermis, Dermis, Subcutaneous, Fascia, Muscle, Bone, Tendon, Ligaments, Deep soft tissue	Tissue damage induced by thermal energy. Removal of dead/necrotic tissue necessary. Prone to infection and bacterial propagation. Severity depends on agent (e.g. flame, electrical, chemical), depth, tissue structures involved, and total area of burn. All burns below dermis are "full thickness," third degree or beyond.	Anywhere	• Vascular and fluid loss • Neurological (Somatic and Autonomic) • Epithelialization • Matrix Deposition • Aggressive Bacterial Invasion • Moisture Control	• Moist semi-permeable or occlusive dressings often treated with silver or other antimicrobial. • Includes gauze, foams, and hydrogels often containing silver. • Skin grafting is common in severe cases.	Must provide moisture to wounds while mitigating bacterial propagation. Non-adherent dressing are preferred. Full thickness burns will require debridement and dressings which aid eschar removal. Early burn fluid loss require extensive use of pads and wraps. Delivery of biological compounds such as stem cells and growth factors have demonstrated the capacity to expedite and improve long term healing outcomes. Hydrogels are of specific interest.	[191–195]
**Radiation Dermatitis**	Epidermis, Dermis, Subcutaneous	Localized skin damaged due to radiation (therapeutic or incidental) results in decreased cellular proliferation and bioactive factor production (e.g. growth factors and cytokines). Damages matrix proteins (e.g. collagen). Shares characteristics with arterial insufficiency.	Anywhere (commonly sites of cancer therapy)	• Cellular proliferation • Epithelialization • Matrix Deposition • Prone to Bacterial Colonization • Increased Sensitivity to Topical Agents	• Saline soaked gauze dressings with silver or other antimicrobial compounds. • Impregnated gauze, hydrocolloid, of hydrogels. • Polyurethane foams and films dressings are also used.	Moisture donating dressings that have anti-radiation factors, such as amifostine, curcumin and corticosteroids, would provide benefits to wound outcomes. Additionally, hyaluronic acid based dressings have demonstrated enhanced wound outcomes. Depth of involvement varies from superficial to deep. Specially formulated, non-sensitizing creams beneficial in superficial radiation burns.	[196–200]
**Penetrating Wound**	Epidermis, Dermis, Subcutaneous, Fascia, Muscle, Bone, Tendon, Ligaments, Deep soft tissue	Wound induced via penetration of an object through the external skin barrier (e.g. gun shot or stab wound). Can be associated with foreign debris and structural damage to soft and hard tissue.	Anywhere	• Depends on tissue involvement • Often vascular and nerve damage can be present.	• Dependent on wound characteristics and tissue involvement. • Packing wounds with gauze and foams is often utilized if wounds are too large to be re-apposed. • Exudate, foreign material and debris removal and moisture control are important. • Film coverings are also common.	Dependent on depth of wound and tissue types involved. Malleable dressing materials and formable gel- and foam- based dressings are ideal for irregular contour of deep penetrating wounds. Hemostatic materials for wounds that continuously bleed to prevent exsanguination.	[201–205]
**Complex Traumatic**	Epidermis, Dermis, Subcutaneous, Fascia, Muscle, Bone, Tendon, Ligaments, Deep soft tissue	Result of traumatic event (e.g. car accident or military-based) where multiple tissues are almost always involved. Can be associated with foreign debris and structural damage to soft and hard tissue, in addition to vascular, nervous, and lymphatic damage.	Anywhere	• Depends on tissue involvement • Often vascular and nerve damage can be present • Lymphatics are also commonly involved with edema typically present	• Dependent on wound characteristics and tissue involvement. • Packing of wounds with gauze and foams or skin grafting can be utilized for larger wounds. • Exudate, foreign material and debris removal and moisture control are important. • Film coverings are also common.	Dependent on complexity of wounds and tissue types involved. Stem cells offer a heterogenous functionality that can aid in the regeneration of multiple tissue types. Malleable dressing materials and formable gel- and foam- based dressings are ideal for irregular contour of complex traumatic wounds. Materials that provide mechanical and dimensional stability ideal for structural damage of tissue. May be paired with negative pressure therapy. Tailoring release of bioactive factors depending on tissue involvement will provide control of tissue regeneration.	[201, 205–207]

^a^Establishment of etiology and the correction of the underlying pathology should be the first order of care. This review looks at dressings presuming that is being carried out.

Benefits of film dressing designs are their adhesive, transparent, and flexible nature. However, they are often limited due to their limited moisture vapor transpiration activity in exudative wounds and mechanical properties, with cracking or tearing upon extreme stretching of the film in some circumstances, in addition to bacterial proliferation within the moisture-trapped wound. Recent studies have demonstrated the ability to improve the antimicrobial properties of films by impregnating antibiotics, silver, silica, or other compounds into the flexible adhesive films dressings [209–211] or fabricating films of bioactive compounds, such as β-glucan paramylon, to enhance their immunomodulatory and wound healing activity [212, 213]. Additionally, the generation of silk-fibroin based films have demonstrated potential benefits of being able to modulate the absorptive and mechanical properties of films, with introduction of a plasticizer (e.g. glucose) further enhancing the flexibility of the silk-fibroin films without compromising the mechanical properties [214] (Fig. 13). Lastly, recent investigations illustrate that conductive film dressings (e.g. polypyrrole nanotubes) have the capacity to promote cellular activity of wound healing cells, such as keratinocytes and fibroblasts [215, 216].

The highly porous nature of foam dressings allow them to absorb large quantities of wound exudate while still maintaining a moist wound environment, compared to the moisture trapping activity of film dressings. However, the trade-off is with more moisture, wounds are more prone to bacterial proliferation. Moreover, the malleable and compressive nature of foams is beneficial, although they typically come as preformed shapes and sizes. Recent studies have investigated the controlled inclusion of antimicrobial compounds, such as antibiotics (e.g. ciprofloxacin), small molecules (antimicrobial peptides), or inorganic compounds (e.g. silver) into foam dressing via covalent or non-covalent linkages [217–221]. Additionally, another interesting clinical study showed how a hydrated polyurethane foam for the delivery of growth factors enhanced the rate of healing in venous leg ulcers [222]. Ultimately, investigations into *in situ* foaming dressings could provide immense benefit, with the ability to apply the dressing as a viscous fluid (e.g. spray, inject, pour, spread) and cure/crosslink within the tissue (Fig. 13). This would enhance the surface contact to the irregular contour of deep, more complex wounds, such as the case often seen with negative pressure wound therapy treated wounds. Similarly, research into the delivery of biological compounds with foam dressings is still very early, but taking advantages of the tunable nature of polymeric biomaterials and the high surface area-to-volume ratio of absorbent foams offers an opportunity for controlled delivery of biomodulatory factors, likely through enzymatic/hydrolytic cleavage of bound compounds or the sustained release via erosion of a polymer substrate.

As research into alginate-based dressings continues, the goal is to better control wound healing responses by reducing inflammation, promoting angiogenesis, improving antimicrobial properties, and enhancing wound moisturization via incorporation of bioactive compounds or blending with other biomaterial based compounds. More specifically, incorporation of chitosan and silver nanoparticles to alginate-based dressings has demonstrated enhanced antimicrobial and immunomodulatory properties [223, 224], whereas incorporation of a sugar moiety or magnesium helped improve the angiogenic potential of alginate dressings [225, 226] (Fig. 13). Similarly, the antimicrobial peptide Tet213 was conjugated to a hybrid alginate/collagen/hyaluronic acid dressing which enhanced the antimicrobial activity against methicillin-resistant Staphylococcus aureus (MRSA) and other bacterial species while maintaining its absorptive, biodegradable, and mechanical properties [227]. Uniquely, is the fact that alginate-derived dressings can come in the form of films, foams, or gels, permitting the customization of alginate dressings for specific wound types and applications, such as the generation of a porous alginate foam embedded with cells and/or biologics that have been encapsulated with an alginate hydrogel.

Hydrocolloids can protect the wound via their semi-permeable or occlusive nature but also contain a colloidal gel layer that helps moisten wound tissue. The colloidal gel layer offers a unique opportunity to modulate the absorptive capacity of the dressing while also delivering a variety of biomodulatory compounds, which is a current area of research. A recent study has demonstrated how a hybrid curdlan-based (glucose-derived polymer) dressing in conjunction with agarose/chitosan resulted in a superabsorbent, non-toxic dressing that did not degrade in the presence of collagenase but could easily be removed without damaging newly deposited tissue [228]. Another study investigated the delivery of platelet-rich fibrin (PRF), which contains a variety of growth factors and cytokines, with different dressings that included a hydrocolloid formulation (Fig. 13). The study found the hydrocolloids resulted in improved control and retainment of the PRF releasate, which they postulated could result in greater enhancement of wound healing activity [229].

Hydrogel based wound dressings historically have been utilized to help donate moisture to wounds, but a drawback to current formulations is their lack of inductive biological cues and that they are prone to infection. Thus, there are a number of ongoing investigations looking into different ways to deliver antimicrobial compounds to wounds with hydrogels. Some antimicrobial formulations include loading the gels with silver and gold nanoparticles [230, 231], incorporating in antibiotics [232–234], or utilizing substrates with inherent antimicrobial properties [235–239]. Antimicrobials can be simply dissolved within the hydrogels, covalently or non-covalently linked to the polymer backbone and/or side-chain, or even microencapsulated within a secondary hydrogel or microparticle (Fig. 13). Additionally, a recent study demonstrated the ability to control the release of antimicrobials via a mechano-responsive hydrogel for more dynamic wound locations [240].

Some of the most commonly studied hydrogels for wound dressings to date are composed of collagen and the glycosaminoglycans (GAGs), hyaluronic acid and chondroitin sulfate [134, 241–243]. Incorporation of GAGs into hydrogels have previously demonstrated the ability to enhance cellular infiltration, proliferation, and spreading [244, 245]. Other naturally-derived polymeric hydrogels include the use of fibrin for its inherent angiogenic properties [246–248], cellulose [249, 250], and alginate or chitosan for their inherent antimicrobial and hemostatic properties [251–253]. In addition to collagen-based peptide hydrogels, are gelatin-based hydrogel. Gelatin is the denatured and chopped up form of collagen peptides and thus maintains many of the same bioactive cues as collagen [254]. Additionally, synthetic modifications to biologically-derived hydrogels are also commonly investigated, such as the use of polyethylene glycol (PEG) to form linker units within collagen or fibrin hydrogels [255, 256]. PEGylating these compounds expands the hydrogel tailorability and permits the conjugation of other peptides, drugs, or other biomodulatory compounds within the hydrogels, as well as the capacity to further modulate the crosslinking kinetics of the gels. Interesting work has also been done recently in the delivery of stem cells to wounds via hydrogel encapsulation and demonstrated benefit in overall wound healing [257–260]. Similarly, encapsulation of stem cell derived exosomes is a new and exciting area of research that demonstrates a novel approach to an acellular regenerative therapy that potentially eliminates to the need for using autologous cell sources [261–263].

The ability to construct hydrogels from both synthetic and naturally occurring compounds and mimic the native fibrous matrix of tissue while permitting fluid and mass transport, highlights some of the benefits of hydrogel-based dressings. Recent investigations have begun to look into utilizing hydrogels as delivery systems for biomodulatory compounds, such as drugs or biologics to promote angiogenesis, re-epithelialization, neotissue formation, and immunomodulation to develop the next generation of tailored wound dressings [264, 265]. Some examples include a hyaluronic acid hydrogel to deliver a DNA plasmid encoding for vascular endothelial growth factor (VEGF) [266], a PVA/chitosan/gelatin hydrogel to deliver FGF-2 [267], a hyaluronan/gelatin hydrogel was used to deliver IL-10 and VEGF-E [268], and a SDF-1 loaded gelatin-based hydrogel [269]. Similarly, other major benefits of hydrogels are the capacity to formulate hydrogels that cure *in situ* or utilization of shear-thinning polymeric hydrogels, allowing for injectable systems [164, 270, 271].

Ultimately, hydrogels potentially offer many advantages as a wound dressing system, including their ability to mimic native tissue microarchitecture, modulate mechanical properties, injectability, mass transport properties, water and moisture content, and ability to easily conjugate and deliver biological factors. Incorporation of hydrogels into other dressing types to form hybrid dressing classes, or as a standalone system, will likely offer superior outcomes in many types of wounds. For example, recent studies have demonstrated how the incorporation of secondary fiber meshes within hydrogels further augment the mechanical properties of hydrogels by forming hybrid fiber/hydrogel composite dressings [272, 273].

Another exciting area of biomaterial research is the generation of smart wound dressings and microneedle patches, such as use of flexible electronics for the continuous monitoring of wound status for bacterial infiltration or biofilm formation, followed by the subsequent ability to locally administer antimicrobials [274–276] (Fig. 13). As technology progresses, these flexible electronic devices may more easily be integrated into current and future wound dressing designs to permit continuous inpatient and outpatient monitoring of wound healing status.

Ultimately, the next generation of wound dressings will likely incorporate biological compounds and deliver those factors in a controlled manner in order to augment the tissue healing process and decrease bacterial burden. Additionally, formulating dressings with customizable macro-properties, such as porosity, permeability, conductivity, and absorption, will aid in maximizing the current capacity of dressings to create a conducive healing environment that is warm, moist, clean, and pH-controlled.

## Future Outlook and Direction

The conception of tissue engineering was a product of the unification of the biological, material, and engineering disciplines. Current and future progress in the field has continued to coalesce a variety of other fields, including computer, electrical, chemical, and biomedical science, with polymer-based materials playing a key role in each of those fields. The diverse utility of synthetic polymers has vastly expanded biomedical therapeutics, with increasing capabilities to fine-tune select polymer characteristics in a predictable and controllable fashion [87]. The incorporation of natural polymers and materials, such as extracellular matrix compounds, polysaccharides, and polypeptides has permitted the further biointegration of polymeric materials into complex native tissue environments. Thus, the field of wound care has greatly benefited from the progress in tissue engineering and has opened the door to a future of more personalized medicine with customized wound dressings that have tailored mechanical, chemical, physical, and biological attributes that enhance both the efficiency and efficacy of wound healing outcomes. Engineering polymeric biomaterials for the controlled delivery of stem cells, biologics, and drugs to augment wound healing and tailor tissue regenerative processes is revolutionizing the development of personalized wound care therapies.

Modern synthesis techniques have expanded the precision and capabilities of fabricating polymer-based materials, with fine-tuned nano-/micro-architecture that more closely mimics the complex fibrous network and mechanics seen in native tissue. Moreover, the evolution of techniques such as self-assembling nanoparticles, “smart” materials, microneedles, piezoelectric materials, organ-on-chip, and electrospinning have garnered a lot of interest for their ability to generate well-defined micro-/macro-biomaterial structures with unique properties. The develop of “smart” materials that respond to specific environmental stimuli (e.g. pressure, temperature, pH, ionic state, magnetism, and electricity) can result in a physiological state-dependent control of cellular behavior. Self-assembling nanoparticles can be produced to form predefined structures, including aligning or crosslinking of polymer fibers (e.g. collagen) to modulate the mechanical properties of the tissue scaffold. Microneedle technology has emerged as a technique that could one day be incorporated into wound dressings for drug delivery applications, or to capture and monitor biochemical profiles, such as cytokines or growth factors, within tissue. The genesis and evolution of electrospinning technology is another area of exciting advancement. The new portable, hand-held polymer extruding gun from Nanomedic opens the door for a new class of customizable wound dressings that architecturally mimic the fibrous nature of native tissue and can be applied in virtually any point-of-care setting, including both surgical and non-surgical environments [277, 278]. Similarly, the advancement of more precise and higher resolution manufacturing techniques, like 3D printing technology, has led to the fabrication of polymeric biomaterials that are more capable of resembling complex tissue structures. For example, preformed vascular and capillary-like channels can be 3D bioprinted within the microarchitecture of a biomaterial graft to improve nutrient exchange and result in more effective integration of synthetic tissue grafts within wound tissue.

The inclusion of different tissue structures, locations, and profiles paired with individual health status and wound type, results in complex wound profiles with individually unique wound healing processes. Thus, as healthcare continues to evolve towards a precision-based medicine model, the diverse, complimentary profile of polymer-based technologies facilitate an exciting approach to potentially treat complex wounds, like burn, traumatic, and chronic wounds. Emerging technologies, such as hydrogel-based dressings, look to mimic the native tissue environment architecturally, mechanically, and biologically more closely, with hopes of generating a wound dressing or graft that promotes more efficient biointegration. Additional inclusion of small molecules, drugs, biologics and cells allows for further promotion of cellular processes with the ultimate goal of wound dressings with customized properties that enhance tissue regeneration and neotissue formation with temporal-spatial control.

## Conclusion

Wound dressings have advanced over the years to include a variety of approaches. However, the same traditional treatment modalities of clean, cover, and moisturize are still used by default. This approach is often effective and adequate for simple acute wounds, but relies solely on the body’s natural capabilities, which is not always adequate in the setting of complex, chronic wounds. Thus, current strategies focus on developing bioactivated wound dressings, where the dressings are used to deliver factors like drugs, biologics, or stem cells, to augment the tissue healing response. Ultimately, combining what we know about the traditional classes of dressing materials to achieve warm, clean, moist, and pH controlled wounds will help to develop a standard “system” of wound dressings rather than one individual dressing type. The next step is figuring out how to customize and tailor these systems towards different wound types, and individual patient populations based on their health or wound status. Thus, the diverse range and adaptability of polymer-based biomaterials paired with the advancement of tissue engineering modalities, appear to play an intimate role in the development of the future of personalized wound care.

## Data Availability

The authors collectively declare that all data supporting the review article are available within the paper and its supplementary information files.
